# A simple novel approach for detecting blood–brain barrier permeability using GPCR internalization

**DOI:** 10.1111/nan.12665

**Published:** 2020-09-27

**Authors:** Z. Csaba, T. Vitalis, C. Charriaut‐Marlangue, I. Margaill, B. Coqueran, P.‐L. Leger, I. Parente, A. Jacquens, L. Titomanlio, C. Constans, C. Demene, M. D. Santin, S. Lehericy, N. Perrière, F. Glacial, S. Auvin, M. Tanter, J.‐F. Ghersi‐Egea, H. Adle‐Biassette, J.‐F. Aubry, P. Gressens, P. Dournaud

**Affiliations:** ^1^ NeuroDiderot Inserm U1141 Université de Paris Paris France; ^2^ Research Team "Pharmacology of Cerebral Circulation" EA4475 Faculté de Pharmacie de Paris Université de Paris Paris France; ^3^ Institut Langevin ESPCI Paris PSL Research University CNRS UMR7587, Inserm U979 Inserm Technology Research Accelerator in Biomedical Ultrasound Université de Paris Paris France; ^4^ Brain and Spine Institute‐ICM Center for NeuroImaging Research – CENIR Sorbonne Paris Cité UPMC Université Paris 06 Inserm U1127 CNRS UMR 7225 Paris France; ^5^ BrainPlotting, Brain and Spine Institute‐ICM Paris France; ^6^ Fluid Team, Lyon Neurosciences Research Center Inserm U1028 CNRS UMR5292 University Lyon‐1 Villeurbanne France; ^7^ Service d'Anatomie et de Cytologie Pathologiques Hôpital Lariboisière APHP Paris France

**Keywords:** blood–brain barrier, cerebral cortex, magnetic resonance imaging (MRI)‐guided focused ultrasound, neurodevelopment, neurological diseases, neurovascular unit, stroke, traumatic brain injury

## Abstract

**Aims:**

Impairment of blood–brain barrier (BBB) is involved in numerous neurological diseases from developmental to aging stages. Reliable imaging of increased BBB permeability is therefore crucial for basic research and preclinical studies. Today, the analysis of extravasation of exogenous dyes is the principal method to study BBB leakage. However, these procedures are challenging to apply in pups and embryos and may appear difficult to interpret. Here we introduce a novel approach based on agonist‐induced internalization of a neuronal G protein‐coupled receptor widely distributed in the mammalian brain, the somatostatin receptor type 2 (SST2).

**Methods:**

The clinically approved SST2 agonist octreotide (1 kDa), when injected intraperitoneally does not cross an intact BBB. At sites of BBB permeability, however, OCT extravasates and induces SST2 internalization from the neuronal membrane into perinuclear compartments. This allows an unambiguous localization of increased BBB permeability by classical immunohistochemical procedures using specific antibodies against the receptor.

**Results:**

We first validated our approach in sensory circumventricular organs which display permissive vascular permeability. Through SST2 internalization, we next monitored BBB opening induced by magnetic resonance imaging‐guided focused ultrasound in murine cerebral cortex. Finally, we proved that after intraperitoneal agonist injection in pregnant mice, SST2 receptor internalization permits analysis of BBB integrity in embryos during brain development.

**Conclusions:**

This approach provides an alternative and simple manner to assess BBB dysfunction and development in different physiological and pathological conditions.

## Graphical abstract

We introduce a novel approach based on agonist‐induced internalization of a neuronal G protein‐coupled receptor widely distributed in the mammalian brain, the somatostatin receptor type 2 (SST2).This approach provides an alternative and simple manner to assess BBB dysfunction and development in different physiological and pathological conditions.

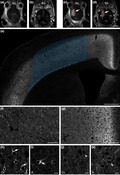

## Introduction

Blood–brain barrier (BBB) dysfunction is a common feature and participates in the aetiology of numerous neurological disorders in adults, including Alzheimer disease, stroke, infections, multiple sclerosis or epilepsy [1, 2, 3]. Aetiological relevance of BBB leakage is also documented in schizophrenia [4] and major depressive disorder [5, 6]. In the developing brain, increasing evidence indicates that BBB disruption contributes to several paediatric neurological conditions [7, 8, 9].

The impressive growing list of neurological diseases involving BBB leakage, both in the developing and the mature brain, deserves accurate, high‐resolution and technically easy *in situ* imaging methods to monitor BBB dysfunction in preclinical models. Today, microscopic examination of exogenous tracer extravasation, such as fluorescent dye‐conjugated dextrans (MW 3–70 kDa) and Evans blue (MW 67 kDa when tied to albumin), remains the main way to assess functional brain microvasculature defects and maturation. Although these technical approaches have considerably improved our knowledge of BBB damage, the use of these tracers has, however, several drawbacks [10, 11, 12]. In particular, the precise delineation of regions with BBB leakage is difficult to define unambiguously, because the extent of diffusion in the brain parenchyma can vary from tracer to tracer, and autofluorescence can limit the sensitivity of the assay. More importantly, tracer injection in the blood flow of embryos (liver or heart) and young rodents requires a technically complex surgical act performed under anaesthesia [13, 14, 15].

To offer an additional technique for functional BBB assessment, we take here a different approach based on ligand‐induced G protein‐coupled receptor (GPCR) internalization. Our simple concept relies on the property of a brain GPCR, the somatostatin type 2 receptor (SST2), to rapidly (within minutes) internalize and concentrate in the perinuclear neuronal compartment after exogenous ligand binding before slowly (over hours) recycling [16, 17, 18]. This receptor, which is only expressed by neurons, is widely distributed in the rodent brain including the cerebral cortex, hippocampus, amygdala, striatum, septum and hypothalamus [19, 20]. The SST2 appears at least as early as E11.5 in mice and remains expressed during rodent brain development [21]. At the regional level when detected by immunohistochemistry, the distribution of nonactivated receptors takes the form of a homogeneous pattern due to localization of the SST2 at the membrane of cell bodies and dendritic arborizations. After activation by an exogenous agonist, receptors internalize and are retrogradely targeted to and concentrated in the *trans*‐Golgi network (TGN) [16, 17, 18]. Such a change in receptor localization is easy to visualize and monitor, even at low microscopic magnification [22, 23]. Because BBB prevents the passage to the brain parenchyma of both proteins and small molecules [24], octreotide (OCT‐sandostatin‐SMS‐201‐995; MW 1 kDa) [25], a clinically approved water‐soluble agonist of this receptor, does not cross the intact BBB. We have therefore postulated that in regions where BBB permeability would be increased due to physiological or pathological conditions, SST2 agonist injected intraperitoneally would enter into the brain from the blood, reach adjacent receptors and provoke their internalization (Figure S1). The mean distance between neuronal nuclei and capillaries (15 µm) guarantees a high efficiency of diffusion‐based agonist exchange between blood and neuronal membrane expressing this receptor [26]. Using complementary models, we demonstrate that this simple approach allows unambiguous characterization of brain areas where the BBB is altered in rodent adults, juveniles and embryos.

## Materials and Methods

### Animals

Experimental procedures were performed using Wistar rats, C57Bl/6 and OF1 mice (Charles River Laboratories, France). All efforts were made to reduce the number of animals used and any distress caused by the procedures is in accordance with the European Communities Council Directive of September 22, 2010 (2010/63/UE) and complying with ARRIVE (https://www.nc3rs.org.uk/arrive‐guidelines) and Inserm guidelines and the ethics committees on animal experiments of Paris Diderot University.

### Antibodies

The endogenous SST2 was immunolocalized using an extensively characterized rabbit monoclonal antibody (1:1000; ab134152, Abcam, Cambridge, UK) [27]. This antibody is directed towards residues 355–369 of the human SST2. This sequence is identical in mouse, rat, and human SST2. See Supporting Information for further details.

#### OCT

OCT (SMS 201‐995; ≥98% HPLC) was purchased from Sigma‐Aldrich (St. Louis, MO, USA).

### In vivo model 1: Circumventricular organs (CVOs) and arcuate nucleus of the hypothalamus (Arc)

*OCT injection*. Post‐natal day 14 (P14) male rats (*n* = 5) were injected with OCT (2.5 mg/kg diluted in 0.01 M phosphate‐buffered saline, pH 7.4 (PBS) i.p.) and were fixed 45 mins later.

### In vivo model 2: Focused ultrasound‐mediated non‐invasive BBB disruption

BBB disruption induced by magnetic resonance imaging (MRI)‐guided focused ultrasound (FUS) (MRgFUS) was performed as previously reported [28]. See Supporting Information for further details.

*OCT injection*. Thirty mins following BBB disruption, mice were injected with OCT (2.5 mg/kg i.p.) and were fixed 45 mins later.

### In vivo model 3: Cerebral ischaemia

*Ischaemia‐reperfusion*. Ischaemia was induced in P14 rats as previously reported [29]. See Supporting Information for further details.

*OCT injection*. Rats 6 h (*n* = 3), 12 h (*n* = 3) or 24 h (*n* = 4) after the ischaemia‐reperfusion procedure were injected with OCT (2.5 mg/kg i.p.) and were fixed 45 mins later.

### In vivo model 4: Traumatic brain injury (TBI)

*Closed weight‐drop head trauma*. TBI was induced in P7 OF1 mice as previously described [30]. See Supporting Information for further details.

*OCT injection*. Mice (n = 3) 30 mins after the traumatic brain injury were injected with OCT (2.5 mg/kg i.p.) and were fixed 45 mins later.

### In vivo model 5: Embryonic BBB permeability

*OCT injection*. Pregnant female C57Bl/6 mice at gestation E13.5 (*n* = 3), E14.5 (*n* = 3), E15.5 (*n* = 3) and E18.5 (*n* = 3) were injected with OCT (2.5 mg/kg i.p.) and were fixed 45 mins later.

### Tissue preparation for in vivo models 1‐5

*Adult mice and post‐natal day 14 (P14) male rats*. Forty‐five mins after OCT injection, animals were deeply anaesthetized with sodium pentobarbital (150 mg/kg i.p.; Ceva Sante Animal, Libourne, France) and perfused through the ascending aorta with 100 mL of 4% paraformaldehyde (PFA) in 0.1 M phosphate buffer, pH 7.4 (PB). Brains were dissected, post‐fixed in the same fixative overnight at 4°C, cryoprotected, frozen in liquid isopentane at −45°C and sectioned in the coronal plane at a thickness of 30 μm and collected in PBS.

*Pregnant female mice and embryos*. Forty‐five mins after OCT injection, dams were deeply anaesthetized with sodium pentobarbital (150 mg/kg i.p.), and embryos were dissected and fixed in 4% PFA in PB overnight at 4°C. The brains of the embryos (n = 5 per age) were cut in the coronal plane on a vibratome at 60 µm and collected in PBS. Additional whole embryos at E15.5 (n = 3) were cut in the sagittal plane to analyse the distribution of SST2 in the pancreas. In parallel, dams were perfused, their brains were post‐fixed, cryoprotected and sectioned as described above.

### *In vitro* model: OCT transport assays through Human Primary Brain Microvascular Endothelial Cell (hPBMEC) monolayers

The hPBMECs were cultured as previously described [31]. See Supporting Information for further details. Differentiated hPBMECs with the BBB phenotype (TEER> 1000 Ωxcm^2^) were treated or not with 1 M mannitol in serum‐free culture medium for 30 min to temporarily open tight junctions. Then, mannitol solution was removed and replaced by culture medium. Tight junction opening was evaluated with the measurements of fluorescein permeability, a small hydrophilic compound known to cross the BBB via the paracellular route. In both mannitol‐treated and untreated hPBMECs monolayers, OCT at 10 μM or 100 μM concentration was added to the luminal compartment. The medium of the abluminal compartment was then collected during a 2‐hour period (*n* = 3 per condition).

To analyse the passage of OCT across the hPBMECs monolayer, the medium collected from the abluminal compartment was then applied to DAOY medulloblastoma cells, which endogenously express SST2. The agonist‐induced SST2 internalization was monitored after cell fixation. DAOY cells were grown in DMEM with 1 g/L D‐Glucose, L‐Glutamine and Pyruvate (31885‐023; Gibco, Life Technologies, Carlsbad, CA, USA) containing 10% foetal bovine serum (FBS; Invitrogen, Life Technologies) and 5000 IU/mL Penicillin/Streptomycin (Invitrogen, Life Technologies). Cells were incubated for 20 min at 37°C with either 1 μM OCT or medium collected from the abluminal compartment of hPBMECs with or without mannitol pre‐treatment. At the end of the incubation period, DAOY cells were fixed with 4% PFA supplemented with 4% sucrose in PB for 20 min at room temperature (RT).

### Immunocytochemistry

The SST2 receptor was immunolocalized on brain sections (*in vivo* models 1‐5), whole embryo sections (*in vivo* model 5) and DAOY cells (*in vitro* model). Brain sections were selected at the level of the circumventricular organs (*in vivo* model 1), at the level of the focused ultrasound‐mediated BBB opening (*in vivo* model 2), at the level of the ischaemia‐reperfusion lesions (*in vivo* model 3), at the level of the traumatic brain injury (*in vivo* model 4) and at the level of the caudal embryonic cortex (*in vivo* model 5). See Supporting Information for further details.

#### Confocal microscopy

Immunofluorescent sections from all *in vivo* and *in vitro* models were analysed using a Leica TCS SP8 confocal scanning system (Leica Microsystems, Wetzlar, Germany) equipped with 405‐nm Diode, 488‐nm Ar, 561‐nm DPSS and 633‐nm HeNe lasers. See Supporting Information for further details.

#### Stereological analysis

Stereological measures of estimated volume in the *in vivo* model 2 was performed with the Volumest plug‐in [32] in the Fiji distribution of ImageJ [33]. Brightfield images of serial coronal sections separated by 180 μm were collected. The number of serial sections, the thickness of the sections, the sampling interval and pixel‐to‐μm scaling were entered into the Volumest plug‐in. Next, the cortical areas displaying BBB disruption detected by SST2 internalization were manually traced and the volume estimate was calculated in the Volumest plug‐in [32].

#### Electron microscopy

Pre‐embedding immunogold immunocytochemistry of SST2 in adult C57Bl/6 mice was performed as previously reported [32]. See Supporting Information for further details.

## Results

### Sensory CVOs

**Figure 1:**
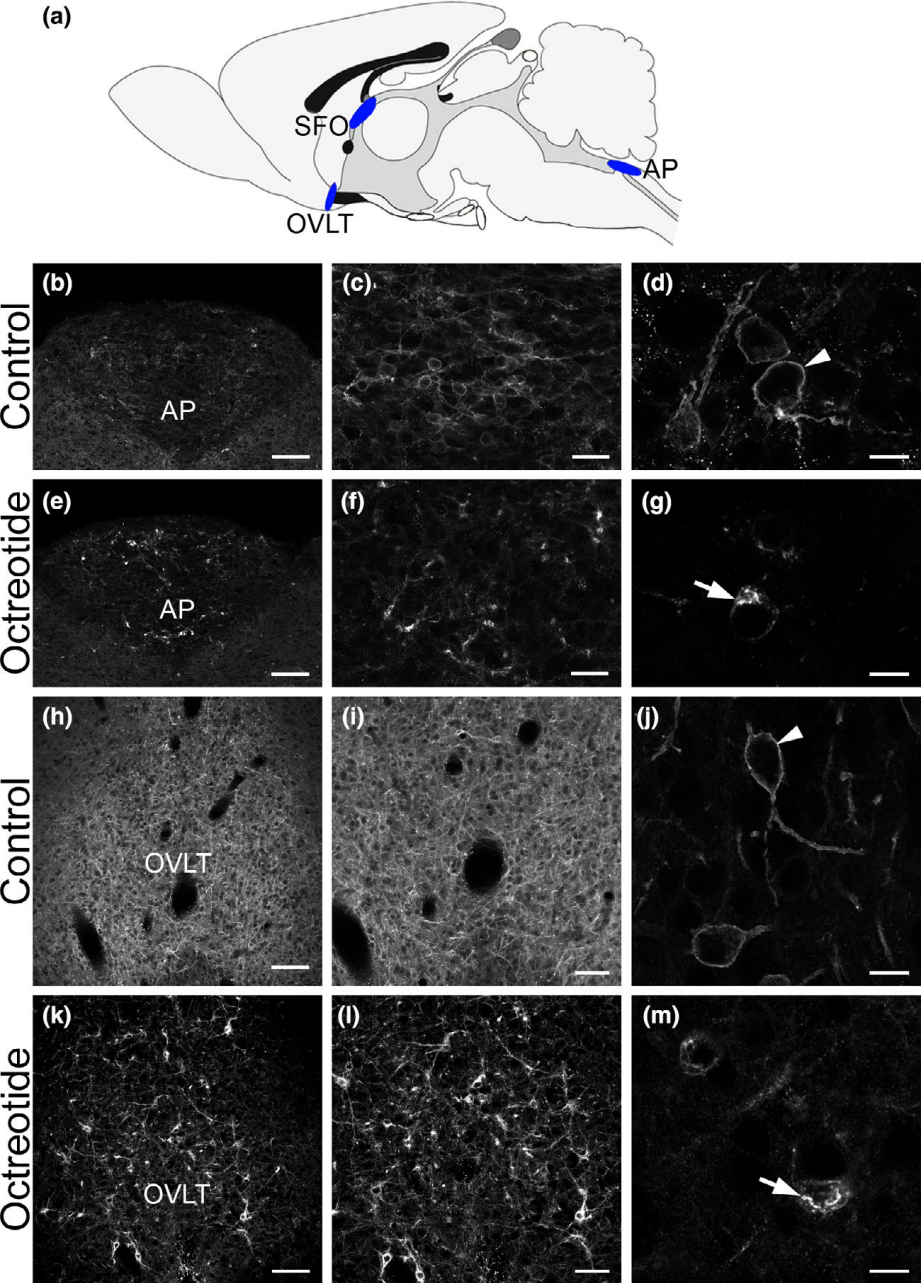
Detection of capillary permeability in sensory circumventricular organs (CVOs): area postrema (AP) and organum vasculosum of the lamina terminalis (OVLT). Schematic localization of CVOs in the rat brain (**a**). At low microscopic magnification, somatostatin receptor 2 (SST2) immunoreactivity appears to fill the entire AP (**b**) and OVLT (h) of control rats. Higher magnification reveals a meshwork of labelled dendrites and neuronal cell bodies (**c**, **i**). At high magnification, SST2 immunoreactivity is localized at the cell surface of neurons in the form of a fluorescent ring both in the AP (**d**) and OVLT (j) (arrowheads) and in a network of dendritic processes. Forty‐five minutes after i.p. injection of SST2 agonist octreotide, a dramatic change of SST2‐immunoreactive pattern can be observed. At the regional level, the homogenous SST2 labelling is no longer evident, SST2 immunoreactivity is concentrated in somatodendritic profiles in the AP (**e**, **f**) and OVLT (**k**, **l**). At the cellular level, SST2 immunoreactivity is confined to bright fluorescent granules in the cytoplasm of neurons in the AP (**g**) and OVLT (m) (arrows), characteristic of agonist‐induced receptor internalization. SFO, subfornical organ. Scale bars: b, e, h, k, 100 μm; c, f, 30 μm; i, l, 60 μm; d, g, j, m, 10 μm.

The sensory CVOs, including the subfornical organ (SFO), organum vasculosum of the lamina terminalis (OVLT), and area postrema (AP) (Figure 1a), receive blood supply through branches of the anterior cerebral (OVLT and SFO) and of the cerebellar (AP) arteries. Sensory CVOs consist of numerous capillary loops that have size‐dependent and function‐related permissive vascular permeability due to the fenestrated nature of their endothelial lining. They allow direct exchange between the blood and the adjacent CNS parenchyma and are critical regulators of cardiovascular function, body fluid balance, immune signalling, temperature control, appetite and reproduction [34].

**Figure 2:**
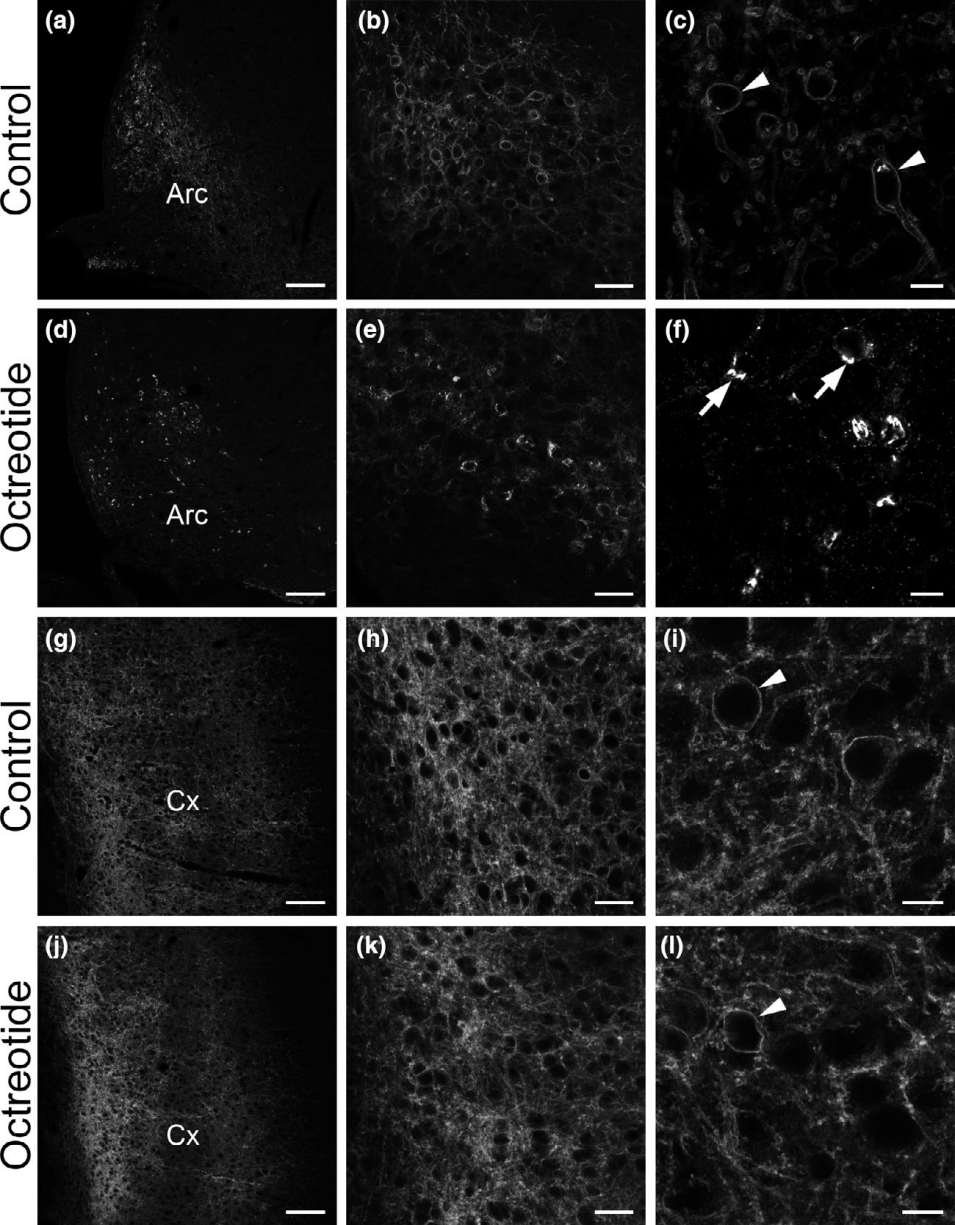
Detection of blood–brain barrier (BBB) permeability in the hypothalamic arcuate nucleus (Arc). At low microscopic magnification, the somatostatin receptor 2 (SST2) immunoreactivity appears to fill the Arc (**a**) and cerebral cortex (Cx; g) in control rats. Higher magnification reveals a meshwork of labelled dendrites and neuronal cell bodies (**b**, **h**). At high magnification, SST2 immunoreactivity is localized at the cell surface of neurons in a form of a fluorescent ring both in the Arc (**c**) and Cx (**i**) (arrowheads) and in a meshwork of dendrites. Forty‐five minutes after i.p. injection of SST2 agonist octreotide (OCT), a change of the SST2 immunoreactive pattern in the Arc can be observed. At low magnification, the homogenous SST2‐immunoreactive pattern is no longer evident and the receptor immunoreactivity is concentrated in somatodendritic profiles (**d**, **e**). At high magnification, SST2 immunoreactivity is confined to bright fluorescent granules in the cytoplasm of Arc neurons (**f**) (arrows), characteristic of agonist‐induced receptor internalization. By contrast, in the Cx, OCT treatment has no effect on SST2‐immunolabelling at the regional (j, k) or cellular (**l**) levels (arrowhead). Scale bars: a, d, g, j, 100 μm; b, e, h, k, 30 μm; c, f, i, l, 10 μm.

Because sensory CVOs are unique areas of the brain where capillaries are permeable, we first tested our working hypothesis in the AP and OVLT, two sensory CVOs in which neurons natively express the SST2. Rats received a single intraperitoneal (i.p.) injection of PBS or OCT (2.5 mg/kg) and were perfused 45 mins after. This dose and time‐point was chosen on the basis of our previous studies and preliminary experiments to allow complete internalization and targeting of activated receptors to the TGN [16, 17, 18]. At low magnification, SST2 immunoreactivity appeared to fill the entire AP and OVLT nuclei in control animals (**Figure** **1b,h**). At higher magnification, serial optical sections using confocal microscopy revealed a meshwork of labelled dendrites and neuronal cell bodies (**Figure** **1c,i**). In both structures, receptor immunoreactivity was predominantly localized at the dendritic and perikaryal plasma membrane (**Figure** **1d,j**), characteristic of nonactivated receptors. In OCT‐injected animals, the homogeneous immunostaining pattern was no longer apparent and neuronal cell bodies with clustered pattern of immunoreactivity were evident (**Figure** **1e,f,k,l**). At high magnification, immunoreactivity was confined to intracytoplasmic granules near the nucleus and in proximal dendrites (**Figure** **1g,m**), a characteristic configuration of activated and internalized receptors. Importantly, in OCT‐injected animals, changes in receptor‐immunoreactive patterns were never observed in regions inside the BBB and adjacent to CVOs such as the nuclei of the horizontal and vertical limb of the diagonal band of Broca (adjacent to OVLT) and nucleus of the solitary tract (adjacent to AP), or distant from CVOs such as the locus coeruleus, the cerebral cortex (**Figure** **2g‐l**) or the hippocampus. These first studies clearly demonstrated that OCT injected in the periphery reached the brain parenchyma only in regions where capillaries were permeable to a 1 k‐Da molecule and induced efficient internalization of the SST2.

### Arcuate nucleus of the hypothalamus

The Arc display several populations of neurons that are pivotal for regulating energy homoeostasis, food intake and cardiovascular functions. Because of this central role at the interface between the brain and the periphery, a few studies have hypothesized that the Arc could share common features with classical CVOs [35, 36]. This issue is, however, still a matter of debate awaiting clear‐cut demonstration. We have therefore reinvestigated this subject using our approach. Following OCT injection, we observed the same results as in the AP and OVLT. Membrane‐associated SST2 in Arc neurons internalized and clustered in TGN‐like structures (**Figure** **2a‐f**). In a previous study using Evans blue, the dye extravasated only in the Arc of 24‐hour fasted mice but not in control‐fed mice [37], whereas our results unambiguously establish that Arc vascular circulation was permeable to at least 1 kDa molecules in naïve animals. This suggests a higher sensitivity of our approach in comparison to Evans blue dye diffusion. Our results thus open the intriguing possibility of a direct passage from blood to Arc neurons of small peptides [38, 39] or cytokines [40].

### Focused ultrasound‐mediated noninvasive BBB disruption

**Figure 3:**
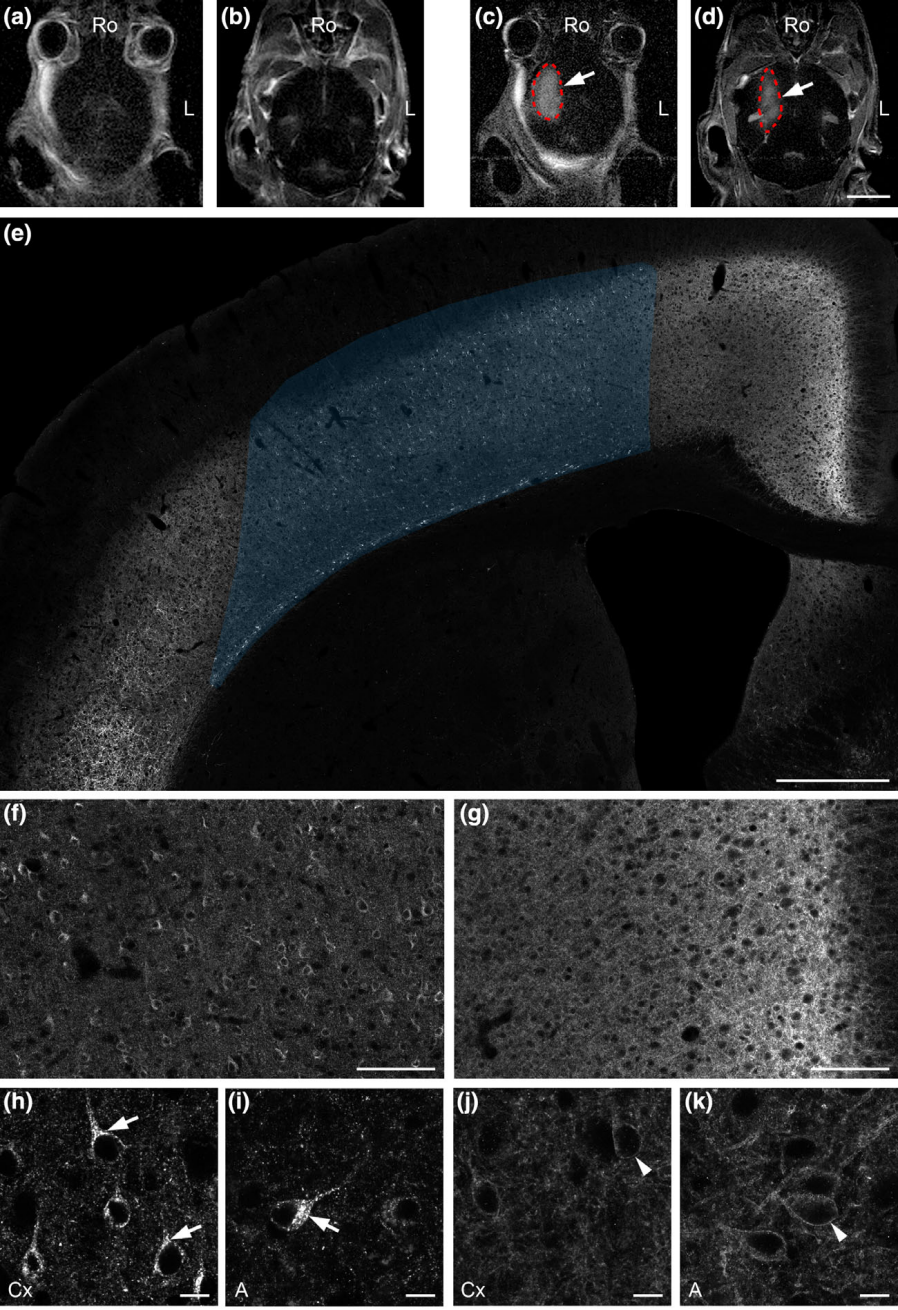
Detection of somatostatin receptor 2 (SST2) agonist extravasation after focused ultrasound‐mediated (MRgFUS) disruption of the blood–brain barrier (BBB) in the mouse brain. Control T1‐weighted MR images close to the dorsal surface (**a**) and in deep brain areas (**b**) obtained before microbubble injection. The gadolinium (Gd)‐based contrast enhancement in the FUS‐targeted area (c, d; red dotted lines) demonstrates opening of the BBB by microbubble sonication (arrows), which extends from dorsal (**c**) to ventral (**d**) brain areas. In the FUS‐targeted right cerebral cortex, clear‐cut area of agonist‐induced SST2 internalization is detected 45 minutes after i.p. injection of SST2 agonist octreotide (shaded area on **e**). Magnified panels show somatodendritic SST2 immunoreactivity in the FUS‐targeted cortex (**f**) as compared to the homogenous SST2 labelling in the surrounding cortex (**g**). At high magnification, agonist‐induced SST2 internalization within the FUS‐targeted area is evidenced by the bright immunofluorescent granules in the cytoplasm (arrows) dorsally in the cortex (Cx; **h**) and ventrally in the amygdala (A; **i**). On the control side, SST2 is located at the surface of neurons both in the Cx (**j**) and in the A (**k**) (arrowheads). Ro, rostral; L, lateral. Scale bars: a‐d, 4 mm; e, 500 μm; f, g, 100 μm; h‐k, 10 μm.

Efficient drug delivery across the BBB into the brain parenchyma remains a major challenge to treating central nervous system disorders for both academic research and the pharmaceutical industry. FUS has recently gained attention for its potential application as a method for locally and transiently disrupting the BBB and thereby facilitating drug delivery into the brain parenchyma [41, 42]. Having demonstrated that we can easily detect brain areas in which BBB was physiologically open, we next investigated whether our technical approach would reveal BBB disruption induced by MRgFUS. We targeted primarily the cerebral cortex, a region with many SST2‐expressing neurons. The highest intensities of SST2 immunolabelling are found in layers 5 and 6, while layers 2 to 4 are less intensely labelled. Using our MRgFUS setup, T1‐weighted (T1w) MRI images demonstrated gadolinium (Gd) extravasation after microbubble sonication of the targeted area. Extravasation was evident from the cerebral cortex to deep brain structures (Figure 3a‐d). There was no visible evidence of haematoma formation or other obvious deleterious consequences of MRgFUS on either MRI studies performed after the BBB disruption or in any of the histological studies. Receptor immunohistochemical staining demonstrated that in the area displaying Gd extravasation on MRI images such as the cerebral cortex dorsally or the amygdala ventrally, SST2 internalization was manifest following peripheral OCT injection (**Figure** **3e,f,h,i**; Videos S1‐2), as previously observed in CVOs and Arc. Clear‐cut borders between areas of internalization and areas where internalization did not occur were evident (Figure 3e). It was therefore easy to estimate the volume of BBB disruption in the deep cortical layers using serial sections (3.59x10^9^ μm^3^; animal 3) (Figure S2). On the contralateral side of the MRgFUS‐targeted region, as well as in other brain areas inside the BBB, SST2 internalization was never observed (**Figure** **3g,j,k**). Of note, we did not observe any change in SST2 distribution or expression at the regional or cellular levels in the brain of PBS‐injected animals following the MRgFUS procedure. Together, these results clearly indicate that a comprehensive mapping of MRgFUS‐BBB opening was possible, thanks to extravasation of the receptor agonist OCT, confirming our expectations. In support of *in vivo* Gd extravasation, it should help to better define at the cellular level the optimum MRgFUS parameters (acoustic pressure, frequency of the transducer, pulse repetition frequency, microbubble size, pulse length) that allow safe, efficient and selective BBB disruption. Testing MRgFUS for BBB opening in larger animal species is a prerequisite for transferring the procedure to human trials. In this context, our approach is also pertinent since the SST2 is expressed in the brain of nonhuman primates and larger animal species [43]. Of note, because the SST2 display both anti‐proliferative and anti‐epileptic properties [43], our MRgFUS noninvasive approach could also be of particular interest to test the therapeutic values of SST2 agonists in preclinical models of brain tumours and epilepsy.

### Cerebral ischaemia

**Figure 4:**
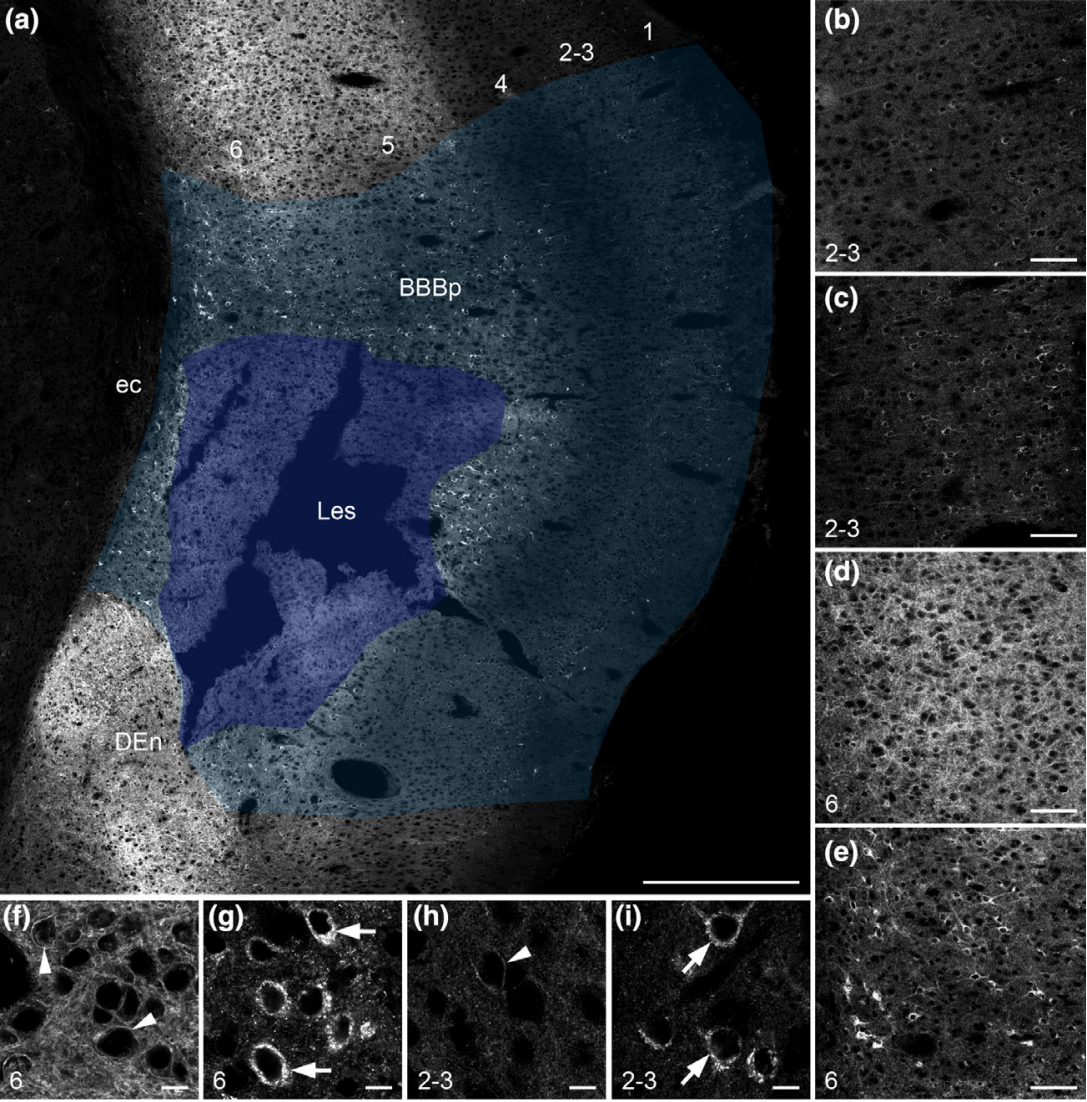
Detection of blood–brain barrier (BBB) permeability in a juvenile cerebral ischaemia rat model. Twenty‐four hours after the cerebral ischaemia, agonist‐induced somatostatin receptor 2 (SST2) internalization is detected in the area of pathological BBB permeability (BBBp; light shaded area on a) around the necrotic cortical lesion (Les; dark shaded area on **a**) 45 minutes after i.p. injection of SST2 agonist octreotide. Magnified panels show somatodendritic SST2 immunoreactivity in the BBBp both in the superficial cortical layers 2–3 (**c**) and in the deep cortical layer 6 (**e**) as compared to the homogenous SST2 labelling surrounding the BBBp in layers 2–3 (**b**) and 6 (**d**). Note that SST2 internalization is evident in both layers 2–3 and 5–6, although the intensity of immunoreactivity is relatively low in the superficial layers as compared to the deep ones. At high microscopic magnification from the BBBp, layer 2–3 (**i**) and layer 6 (**g**) neurons are characterized by the bright intracytoplasmic granules (arrows) as compared to the surface labelling of neurons in layers 2–3 (**h**) and layer 6 (**f**) in the intact cortex. DEn, dorsal endopiriform nucleus; ec, external capsule. Scale bars: a, 500 μm; b‐e, 100 μm; f‐i, 10 μm.

**Figure 5:**
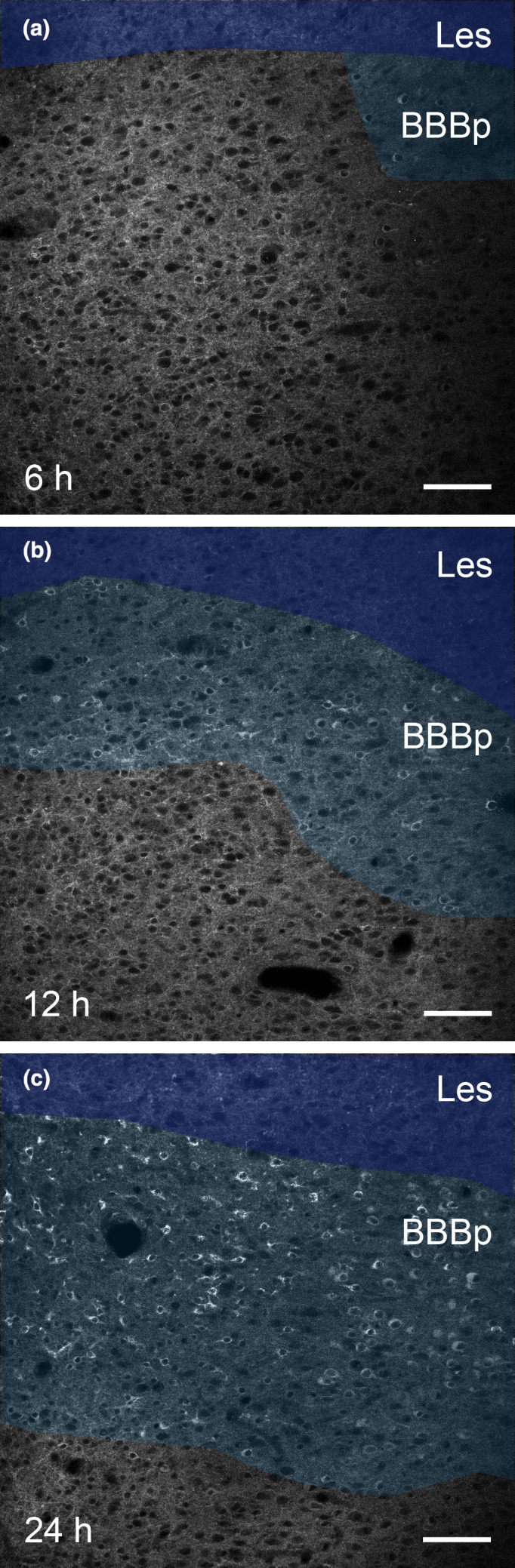
Change of blood–brain barrier (BBB) permeability in a juvenile cerebral ischaemia rat model. Extravasation of i.p. injected somatostatin receptor 2 (SST2) agonist octreotide was monitored 6 h (**a**), 12 h (**b**) and 24 h (**c**) after the initial cerebral ischaemia. Six hours after the cerebral ischaemia, only few neurons with internalized SST2 are detected in the area of pathological BBB permeability (BBBp; light shaded area on a) adjacent to the necrotic cortical lesion (Les; dark shaded area on a). Twelve hours after the cerebral ischaemia, a larger number of neurons with internalized SST2 are localized in a narrow band (light shaded area on b) around the cortical lesion (dark shaded area on b). The BBBp with neurons of internalized SST2 becomes wider 24 h after the cerebral ischaemia (light shaded area on c) around the cortical lesion (dark shaded area on c). Scale bars: a‐c, 100 μm.

Arterial ischaemic stroke is increasingly recognized as a serious paediatric problem. Groups at risk are new‐borns (the first 28 days of life), especially full‐term infants and older children with sickle cell anaemia or congenital heart defects [44]. Neonatal stroke produces significant morbidity and severe long‐term neurological and cognitive deficits, including cerebral palsy, epilepsy, neurodevelopmental disabilities, impaired vision and language function and emotional symptoms. There is a large body of work demonstrating disturbances of BBB function following hypoxia and/or ischaemia in adult animal models [45]. Surprisingly, very few studies focused on the developing brain, at least in part due to some difficulties of accurately assessing BBB integrity in pups using available technical approaches. Because some [46, 47, 48], but not all [15], of these studies do indicate that barrier function is disturbed during ischaemic insults in neonates and juveniles, we investigated whether our methodological approach could reveal BBB leakage in a P14 rat ischaemic model. At 24 h after the initial insult, peripheral OCT injection induced large areas of SST2 internalization around the core of the cortical lesion (Figure 4a). Neurons expressing internalized receptors were visible in cortical layer 2–layer 6, in accordance with the fact that the core of the infarct is located in deep and mediocortical layers [49] (**Figure** **4b‐i**). Again, rapid examination of the sections allowed easy differentiation between regions where internalization occurred and regions where internalization did not. We next monitored the cortical neurons expressing internalized receptors, 6, 12 and 24 h after stroke in comparable areas adjacent to the core of the lesion. Although few neurons with internalized receptors were visible at 6 h after stroke (**Figure** **5a**), the area containing neurons with internalized SST2 became progressively wider (**Figure** **5b,c**). Such a result strongly suggests that areas of receptor internalization are not due to passive diffusion of OCT within the brain parenchyma but represent time‐dependent BBB disruption after the ischaemic insult. Of note, we did not observe any change in SST2 distribution or expression at the regional or cellular levels in PBS‐injected ischaemic animals, except for a loss of receptor immunoreactivity in the necrotic core due to neuronal cell death. Our approach is therefore appropriate to easily and accurately monitor damage of the BBB in adult but also in juvenile ischaemic stroke models.

### Traumatic brain injury

**Figure 6:**
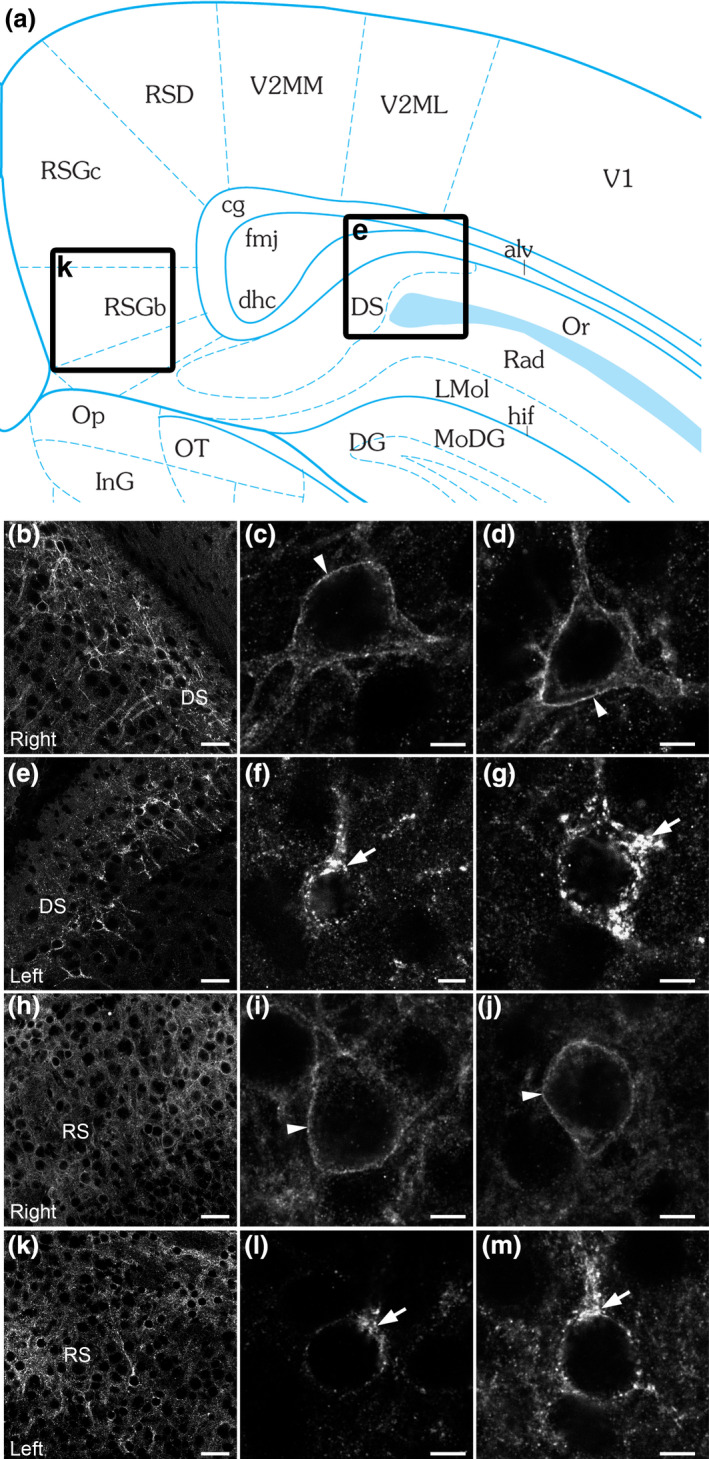
Detection of blood–brain barrier (BBB) permeability in a juvenile traumatic brain injury (TBI) model. Schematic of the mouse brain regions at the level of the contusion impact (**a**). Thirty minutes after the TBI, agonist‐induced somatostatin receptor 2 (SST2) internalization is detected in neurons of the dorsal subiculum (DS; **e**) and retrosplenial cortex (RS; **k**). At high microscopic magnification, neurons are characterized by the bright intracytoplasmic granules in the DS (**f**, **g**) and RS (**l**, **m**) (arrows) 45 min after i.p. injection of SST2 agonist octreotide. On the contralateral side of the contusion, by contrast, the SST2 immunoreactivity is homogeneously distributed in the DS (**b**) and RS (**h**). At high magnification, SST2 immunoreactivity is localized at the cell surface of neurons in the DS (**c**, **d**) and RS (**i**, **j**) (arrowheads). Scale bars: b, e, h, k, 30 μm; c, d, f, g, i, j, l, m, 5 μm.

The consequences of head trauma are also a serious paediatric problem [50]. Deciphering the pathophysiology of the increased susceptibility of the immature brain is therefore of critical importance. Moreover, children under 4 years of age suffer TBI more often than any other age group. Despite its paediatric importance, the accurate assessment of BBB integrity following head injury, which was widely studied in adult animal models [51], is lacking in pups, at least partly because of technical difficulties with available approaches. Because our approach is particularly suitable in pups, we studied whether BBB disruption following TBI could be detected in mouse pups. Thirty minutes after TBI, SST2 internalization was evident only in a limited number of neurons following systemic OCT administration. They were localized on the left side at the rostrocaudal level of the contusion impact (**Figure** **6a**) in the dentate gyrus, subiculum (**Figure** **6e‐g**) and retrosplenial cortex (**Figure** **6k‐m**). The dorsal cortical areas directly beneath the skull at the level of the impact did not display agonist‐induced SST2 internalization. On the contralateral side of the TBI, SST2 internalization was never observed (**Figure 6b‐d,h‐j**). These results demonstrate for the first time that TBI induces early BBB alterations in particular regions of the juvenile brain, opening the way to detailed analyses of the short‐ and long‐term consequences of such alterations, including inflammation, neuronal cell death or connectivity.

### Cellular and subcellular localization of SST2 in the murine brain

Double‐labelling experiments at the light microscopic level confirmed that SST2s are internalized only in neurons but not in astrocytes, microglial cells or endothelial cells (Figures S3,S4a‐c). Moreover, pre‐embedding immunogold immunocytochemistry demonstrated at the ultrastructural level that SST2 immunoparticles were associated with neuronal perikarya and dendrites. In agreement with the C‐terminal epitope of the antibody, SST2 immunoparticles were localized at the plasma membrane outlining the internal side (Figure S4d‐f) or in endosomes outlining the external side (Figure S4g). It is of note that neither glial nor endothelial cells displayed any SST2 immunogold staining (Figure S4d‐g) in agreement with previous studies [16, 17, 53].

### Embryonic BBB permeability

**Figure 7:**
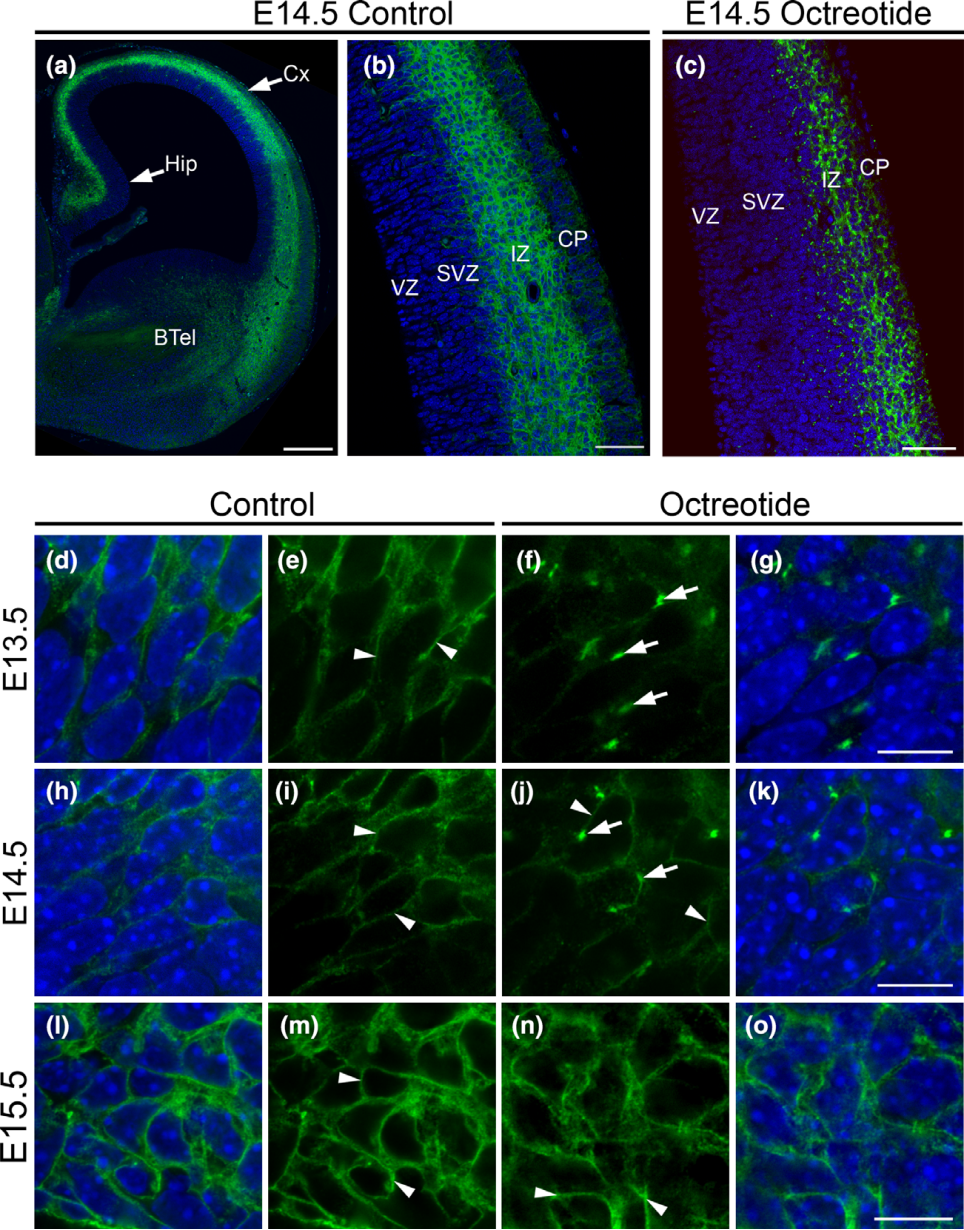
Analysis of blood–brain barrier (BBB) permeability in E13.5‐E15.5 embryos. In a coronal section of an E14.5 control embryo, somatostatin receptor 2 (SST2) immunoreactivity is localized in the hippocampus (Hip), cerebral cortex (Cx) and in the basal telencephalon (BTel) (**a**). In the Cx of controls, a meshwork of SST2 immunolabelling is intense in the intermediate zone (IZ) and weak in the cortical plate (CP). Few SST2‐immunolabelled radial processes are also detected in the ventricular zone (VZ) and subventricular zone (SVZ) (**b**). In an E14.5 embryo of an octreotide (OCT)‐treated dam, the SST2‐immunoreactive pattern is drastically modified and appears as intense immunoreactive puncta in the IZ and CP (**c**). At high microscopic magnifications from the IZ of E13.5 (**d**, **e**), E14.5 (**h**, **i**) and E15.5 (**l**, **m**) control embryos, SST2 immunolabelling is at the cell surface of neurons and in a network of neuritic processes (arrowheads). By contrast, in an E13.5 embryo of an OCT‐treated dam (**f**, **g**), SST2‐immunostaining in the IZ is confined to bright fluorescent granules in the perinuclear compartment (arrows), characteristic of agonist‐induced receptor internalization. Note that in an E14.5 embryo of an OCT‐treaded dam (**j**, **k**), the SST2 immunolabelling in the IZ is partly intracellular (arrows) but also at the cell surface (arrowheads). In an E15.5 embryo of an OCT‐treated dam (**n**, **o**), SST2 immunoreactivity in the IZ is predominantly localized at the cell surface (arrowheads) similarly to controls. Nuclei are labelled with DAPI. Scale bars: a, 350 μm; b, c, 65 μm; d‐o, 10 μm.

From a clinical point of view, it is of particular importance to understand the physiological properties of the BBB during normal and pathological development. On the one hand, such knowledge would help better understand whether and how drugs and toxins entering the foetal circulation from the mother would impact the developing brain. On the other hand, BBB disruption could provide therapeutic windows to deliver neuroprotective molecules in the context of early brain insults. Although molecular and mechanistic insights of BBB development is gradually being discovered [2, 52], the developmental regulation of BBB permeability still represents a major issue. To our knowledge, only few studies have recently set up this challenge and identified the role of key molecular players in functional BBB maturation, Mfsd2a [14] and LSR/angulin‐1 [13]. In the former, a 1–5 µl solution of lysine fixable 10 kDa dextran‐tetramethylrhodamine (TRITC) was injected in the liver of embryos (E13.5 to E15.5) still attached via the umbilical cord to the anaesthetized dams. In the latter, after caesarean section of anaesthetized pregnant females, embryos (E13.5 to E16.5) dissected out from yolk sacs, but still attached to the placenta, were injected into the heart with a solution of 6 µl TRITC‐conjugated 10 kDa dextran or Sulfo‐NHS‐biotin. Both studies, in agreement with pioneer studies by Daneman and colleagues [54], concluded that dyes stop leaking in the cerebral cortex between E14.5 [13] and E15.5 [14]. Clinical studies have reported transplacental passage of OCT by passive diffusion without detectable modification during the maternal–foetal transfer, nor adverse effects to the foetus [55, 56]. Because SST2 appears at least as early as E11.5 in mice and remains expressed during rodent brain development (**Figure** **7a‐c**) [21], we investigated whether i.p. OCT injection in pregnant dams can reveal dynamics of BBB maturation in E13.5 to E18.5 embryos. OCT injection of pregnant females indeed induced a massive redistribution of the receptor from the membrane to intracellular compartments in the cortex of E13.5 embryos (**Figure** **7d‐g**). At E14.5, internalization was still clearly visible although in contrast to earlier ages, membrane‐associated receptors were present **(Figure 7a‐c,h‐k)**. At E15.5 and after, while SST2 internalization was observed in the periphery such as in the pancreas (Figure S5), it was no longer observed in the cerebral cortex (**Figure** **7l‐o**) demonstrating that BBB was sealed to molecules> 1 kDa, in agreement with the studies discussed above. Together, our results demonstrated that this simple approach, which avoids dam anaesthesia, caesarean section and embryo manipulation, allowed the spatial and temporal monitoring of functional BBB maturation across brain development. It should also help to analyse the consequences of different maternal health status (i.e. infectious, nutritional, toxicologic) and genetic manipulations of key proteins of BBB integrity and maturation.

### *In vitro* octreotide transport assay through hPBMECs

**Figure 8:**
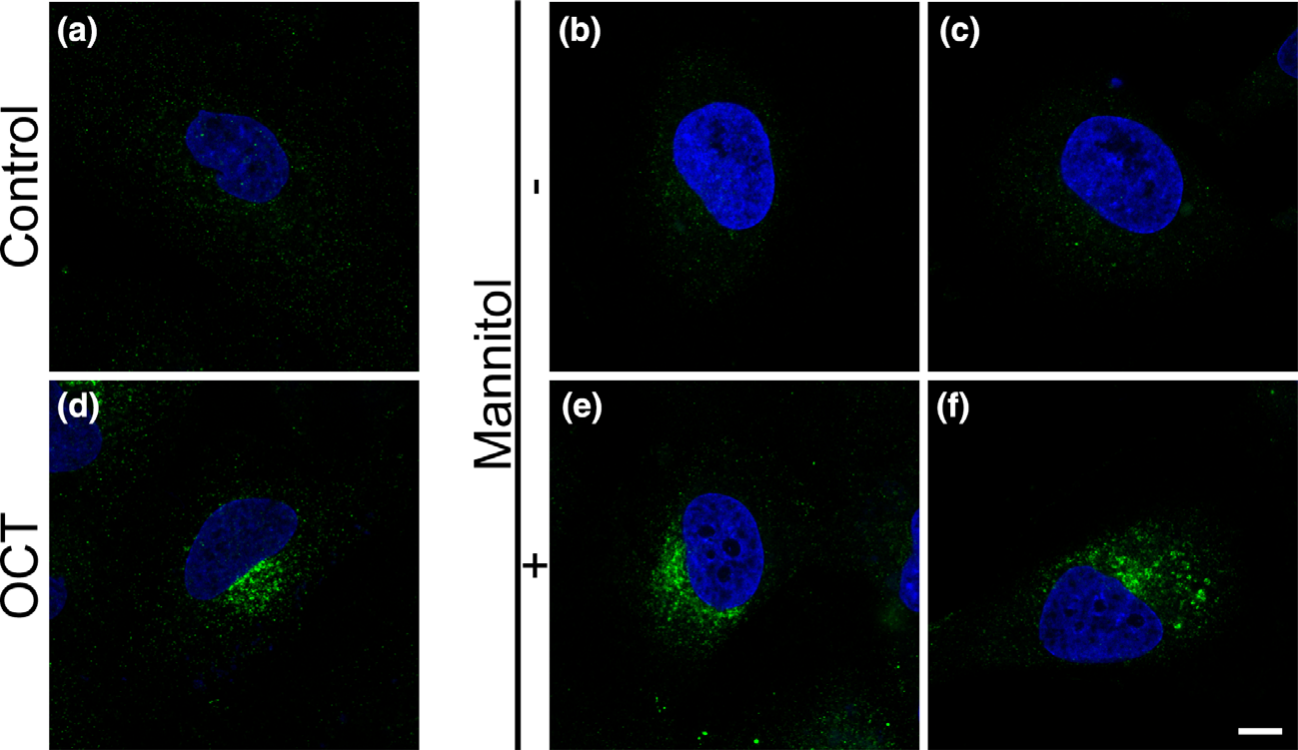
Detection of octreotide transport through Human Primary Brain Microvascular Endothelial Cells (hPBMECs). In a control DAOY cell, somatostatin receptor 2 **(**SST2) immunoreactivity is evenly distributed over the cellular surface (**a**). Similarly, SST2 immunoreactivity in cells treated for 20 mins with the abluminal medium of hPBMECs without mannitol pretreatment is also evenly distributed on the surface, with both 10 μM (**b**) or 100 μM (**c**) luminal octreotide (OCT) concentrations. Treatment with 1 μM OCT for 20 mins results in a massive redistribution of SST2 immunoreactivity into bright fluorescent intracytoplasmic granules (**d**). Similarly, SST2 immunoreactivity in cells treated for 20 mins with the abluminal medium of hPBMECs following mannitol pretreatment is also confined to bright fluorescent granules, with both 10 μM (**e**) or 100 μM (**f**) luminal OCT concentrations. Nuclei are labelled with DAPI. Scale bars: a‐f, 10 μm.

Although OCT does not cross an intact BBB, previous studies have demonstrated that OCT or somatostatin analogues can cross the endothelial monolayer via the paracellular route when tight junctions become loose [57, 58, 59]. This is in accordance with our results obtained by MRgFUS‐BBB opening. This method utilizes the mechanical effect of microbubble oscillations which induced a transient disruption or loosening of the tight junctions in the brain endothelial cells thus facilitating paracellular permeability [41]. In addition, we have conducted experiments using an *in vitro* model of adult human BBB to monitor passage of OCT through the brain endothelial monolayer by agonist‐induced receptor internalization. Using a hypertonic concentration of mannitol, we induced a rapid and reversible increase in hPBMECs permeability to small hydrophilic molecules through the alteration of tight junction complexes [60]. As control, TEER drastically fell with Mannitol treatment (< 20 Ωxcm^2^), and the functionality of the tight junctions was measured in each insert: with the treatment of mannitol, the fluorescein permeability showed an increase of 600 ± 25% (0.135x10^‐3^ cm/min in basal conditions). We assessed permeability of 10 and 100 µM OCT from the luminal side to the abluminal side in control conditions *vs*. after mannitol treatment. After an incubation of 2 h, abluminal media was collected and applied to DAOY cells that endogenously express the SST2 receptor. In control conditions, the distribution of SST2 in DAOY cells was restricted to the cell membrane (**Figure** **8a‐c**). By contrast, incubation of the abluminal media obtained following mannitol pretreatment induced a massive internalization of SST2 in DAOY cells (**Figure** **8d‐f**). Taken together, these results indicate that OCT crosses the endothelial cell monolayer by paracellular transport only when tight junctions are widened.

## Discussion

BBB dysfunction is increasingly recognized as being part of the aetiologic processes that drive a wide range of neurological disorders. Reliable and reproducible approaches are therefore mandatory to accurately monitor BBB disruption in animal models. Visualization of BBB permeability is classically performed using injection of Evans blue or labelled dyes into the blood flow [10, 11, 12, 61]. However, modes of injection require trained and skilled personnel and are challenging to establish in pups and embryos. Animal perfusion as well as brain tissue manipulation have to be carefully monitored to ensure reliable interpretation.

In the present study, we propose and assess a novel alternative method for rapid and precise evaluation of animal BBB disruption and development. The major strength of this method is that a single intraperitoneal injection of a somatostatin analogue (octreotide) induces an easily detectable receptor internalization restricted to the region of BBB leakage.

### Redistribution of SST2 labelling

After agonist binding, membrane‐associated SST2s internalize and concentrate in the perinuclear neuronal compartment [16, 17, 18]. Such a process avoids any concern relating to the detection of dye extravasation. In the different models we have analysed, the clear‐cut difference between neurons that internalized the SST2s and those which did not circumvent any misleading interpretation. SST2 receptor targeting to the TGN after internalization is only observed at pharmacological doses. Such doses induce an acute and massive receptor activation that synchronizes internalization, trafficking and targeting of the receptors to the TGN. After physiological or pathophysiological activation by endogenous ligand, only a few vesicles resembling endosomes bearing SST2 receptor can be visualized in dendrites and/or the neuronal cytoplasm [16, 17]. Such labelling cannot be confused with the massive localization of the receptors in the TGN induced by an exogenous ligand. In addition, because of the high densities of neurons expressing this receptor, delineation of the area with BBB leakage with our method is highly accurate.

### Animal and tissue manipulations

Another key advantage of this approach concerns animal and brain tissue manipulation. In contrast to classical dye injection methods, the receptor agonist injections are done intraperitoneally. This limits animal stress associated with experimental procedures and avoids anaesthesia which can be source of changes in BBB permeability [62]. Such a route of injection is particularly beneficial for BBB studies in pups. Because of the transplacental passage of OCT, this method also offers a simple way to study BBB in embryos.

### Detection of BBB leakage

Detection of BBB leakage through SST2 internalization is realized by classical immunohistochemical procedures, which are routinely performed in research laboratories, and excellent antibodies are commercially available. Neither perfusion of the animals nor brain tissue manipulation can influence the pattern of receptor localization. The clear‐cut identification of BBB leakage by standard immunohistological procedures makes quantification simple and accurate. The regional and cellular distribution of this somatostatin receptor have been indeed particularly documented [16, 17, 18]. In addition, because SST2 detection is compatible with both paraformaldehyde and glutaraldehyde fixations, co‐localization of different proteins of interest at the BBB, by light or electron microscopy, can be realized in the same brain samples. Of note, the SST2 is not only present in the rodent brain but also in larger mammal species including primates [63, 64, 65]. Our approach is therefore suitable to study BBB integrity in different clinically relevant experimental models.

### Advantages of the SST2 agonist

To induce SST2 internalization we chose the commercially available SST2 agonist OCT, which display nM affinity range for this receptor. OCT is a cyclic octapeptide which was approved by the US Food and Drug Administration (FDA) in October 1988 for treatment of acromegaly, treatment of diarrhoea and flushing in patients with metastatic carcinoid tumours [66]. It is still the most widely used somatostatin analogue for treatment of acromegaly, neuroendocrine tumours and gastrointestinal disorders in adult and in paediatric patients. Its primary advantages over somatostatin are a longer half‐life (1.7–1.9 h *vs*. 2–3 mins) in the circulation, a higher potency, and good bioavailability. In humans, OCT is well tolerated with only minor possible side effects such as nausea and diarrhoea. In our rat and mice models, we did not observe any gross changes in physiological or behavioural parameters after acute OCT i.p. injection until animal perfusion, highlighting the interest of the use of OCT in our approach.

### Assessment of OCT passage

Our results obtained by MRgFUS‐BBB opening indicated a paracellular route of OCT passage into the brain. Indeed, this method utilizes the mechanical effect of microbubble oscillations which induced a transient disruption or loosening of the tight junctions in the brain endothelial cells thus facilitating paracellular permeability [41]. In addition, our *in vitro* model of human BBB also demonstrated the crossing of OCT through the endothelial monolayer by paracellular transport, in accordance with previous studies [57, 58, 59]. The juvenile cerebral ischaemia model, however, argues in favour of OCT being able to enter the brain through transcellular vesicular passage. Previous studies have demonstrated that transcytosis is increased in endothelial cells after stroke while the tight junctions remain generally intact during the first 24 h poststroke [67, 68].

### Regions of interest and limitations

Strong SST2 labelling is detected in the deep layers of the cerebral cortex, CA1 field and dentate gyrus of the hippocampus, lateral septum, medial septum/diagonal band of Broca, medial habenula, bed nucleus of the stria terminalis, endopiriform nucleus, claustrum, amygdaloid complex, arcuate nucleus, locus coeruleus and nucleus tractus solitarius [19, 20, 69]. Even in regions where the density of neurons expressing the SST2 receptor is lower (such as medial preoptic area or periventricular nucleus of the hypothalamus), following agonist‐induced internalization in individual neurons (cell bodies and proximal dendrites) is always highly immunoreactive and easily detectable by classical immunohistochemical procedures [22]. This phenomenon is explained by the fact that internalization induces intracellular concentration of receptors which increases the immunohistochemical signal detection. Although the SST2 is distributed in key brain structures for BBB research (such as the cerebral cortex, amygdala, striatum or hypothalamus: for a description see [19, 20]), one limitation of our method is that this GPCR receptor is absent in some brain regions (such as cortical layer 1, dorsal thalamus or white matter). This method might, however, be suitable for other GPCRs that undergo ligand‐induced internalization and are expressed in a particular region of interest. In such cases, receptor agonist should be chosen on the basis of their binding properties, aqueous solubility and molecular weight.

In conclusion, we have introduced a novel approach to study BBB integrity in embryo, juvenile and adult preclinical rodent models. Because of the high sensitivity and simplicity of our method, we anticipate that it could provide new opportunities for both pharmaceutical and academic laboratories to study BBB leakage in different pathological conditions, and to test the efficacy of various therapeutic strategies to protect or to open the BBB, especially during development.

## Author contributions

Z.C. and T.V. designed, performed and interpreted experiments, prepared the figures, and contributed to the writing of the paper. C.C‐M., I.M., B.C., P‐L.L., I.P. designed, performed and interpreted experiments of the cerebral ischaemia model, C.C‐M. also contributed to the writing of the paper. C.C., M.D.S., S.L., C.D., and J‐F.A. designed, performed and interpreted experiments of the focused ultrasound‐mediated BBB opening and contributed to the writing of the paper. A.J. designed, performed and interpreted experiments of the traumatic brain injury. N.P. F.G. designed, performed and interpreted experiments of the *in vitro* BBB model. L.T., S.A., M.T., J‐F.G‐E., H.A‐B., J‐F.A., P.G. were involved in the study design and interpretation of results. P.D. conceived and supervised the project, prepared the figures and wrote the paper.

## Conflict of interest

The authors declare that they have no competing interests.

## Ethics approval

The study was approved with permission from Inserm guidelines and the ethics committees on animal experiments of Paris Diderot University.

## Consent for publication

All authors have read and approved the manuscript and agreed to publish in this journal.

### Peer Review

The peer review history for this article is available at https://publons.com/publon/10.1111/nan.12665.

## Supporting information


**Data S1**. SI Materials and Methods
**Figure S1**. Schematic representation of detection of increased blood‐brain barrier (BBB) permeability using ligand‐induced somatostatin receptor 2 (SST2) internalization.
**Figure S2**. Quantification of somatostatin receptor 2 (SST2) agonist extravasation after focused ultrasound‐mediated (MRgFUS) disruption of the blood‐brain barrier (BBB) in the cerebral cortex.
**Figure S3**. Characterization of cells with internalized somatostatin receptor 2 (SST2) following SST2 agonist extravasation in the rodent brain.
**Figure S4**. Characterization of somatostatin receptor 2 (SST2) localization in relation to blood vessels in the rodent brain.
**Figure S5**. Confocal microscopic analysis of somatostatin receptor 2 (SST2) immunoreactivity in the periphery of E15.5 embryos following SST2 agonist injection of the dams.

**Video S1**. Low magnification 3D reconstruction of somatostatin receptor 2 (SST2) immunoreactivity in the mouse cerebral cortex following focused ultrasound‐mediated (MRgFUS) opening of the blood‐brain barrier (BBB).

**Video S2**. High magnification 3D reconstruction of somatostatin receptor 2 (SST2) immunoreactivity in the mouse cerebral cortex following focused ultrasound‐mediated (MRgFUS) opening of the blood‐brain barrier (BBB).

## Data Availability

The data that support the findings of this study are available from the corresponding author upon reasonable request.
