# Supplementary material for: A simple novel approach for detecting blood–brain barrier permeability using GPCR internalization
Source: Neuropathol Appl Neurobiol. 2020 Sep 27;47(2):297–315. doi: 10.1111/nan.12665 (PMC7891648; doi:10.1111/nan.12665)
Supplement: Supplementary file 1 — Data S1. SI Materials and Methods Figure S1. Schematic representation of detection of increased blood‐brain barrier (BBB) permeability using ligand‐induced somatostatin receptor 2 (SST2) internalization. Figure S2. Quantification of somatostatin receptor 2 (SST2) agonist extravasation after focused ultrasound‐mediated (MRgFUS) disruption of the blood‐brain barrier (BBB) in the cerebral cortex. Figure S3. Characterization of cells with internalized somatostatin receptor 2 (SST2) following SST2 agonist extravasation in the rodent brain. Figure S4. Characterization of somatostatin receptor 2 (SST2) localization in relation to blood vessels in the rodent brain. Figure S5. Confocal microscopic analysis of somatostatin receptor 2 (SST2) immunoreactivity in the periphery of E15.5 embryos following SST2 agonist injection of the dams. [file NAN-47-297-s001.docx]

Supporting Information for:

**A simple novel approach for detecting blood-brain barrier permeability using GPCR internalization**

Zsolt Csaba^1#^, Tania Vitalis^1#^, Christiane Charriaut-Marlangue^1^, Isabelle Margaill^2^, Bérard Coqueran^2^, Pierre-Louis Leger^1^, Iolanda Parente^1^, Alice Jacquens^1^, Luigi Titomanlio^1^, Charlotte Constans^3^, Charlie Demene^3^, Mathieu D. Santin^4^, Stéphane Lehericy^4^, Nicolas Perrière^5^, Fabienne Glacial^5^, Stéphane Auvin^1^, Mickaël Tanter^3^, Jean-François Ghersi-Egea^6^, Homa Adle-Biassette^1,7^, Jean-François Aubry^3^, Pierre Gressens^1^ and Pascal Dournaud^1^*

Pascal Dournaud, PhD

Email: pascal.dournaud@inserm.fr

**This file includes:**

SI Materials and Methods

Figs. S1 to S5

Captions for Video S1 and Video S2

**SI Materials and Methods**

***Antibodies.*** In addition to the rabbit monoclonal anti-SST2 antibody, other primary antibodies used were a mouse monoclonal anti-NeuN antibody (1:1000; MAB377, Merck Millipore, Burlington, MA, USA), a mouse monoclonal anti-GFAP antibody (1:2000; G3893, Sigma-Aldrich, St. Louis, MO, USA), a goat polyclonal anti-Iba1 antibody (1:2000; Ab5076, Abcam, Cambridge, UK), and a rabbit polyclonal anti-GLUT-1 antibody (1:500; 07-1401, Merck Millipore). Secondary antibodies used were Alexa Fluor 488 (A488)-conjugated donkey anti-rabbit (1:200; Life Technologies, Molecular Probes) or biotinylated goat anti-rabbit (1:300; Vector Laboratories, Burlingame, CA, USA) to immunolocalize SST2. Cyanine 3 (Cy3)-conjugated donkey anti-mouse (1:300; Jackson ImmunoResearch Laboratories, West Grove, PA, USA), Cy3-conjugated donkey anti-goat (1:300; Jackson ImmunoResearch Laboratories), and Cy5-conjugated donkey anti-rabbit (1:300; Jackson ImmunoResearch Laboratories) antibodies were used to immunolocalize NeuN and GFAP, Iba1, and GLUT-1, respectively.

***Focused ultrasound-mediated non-invasive BBB disruption***

*Ultrasonic BBB disruption system.* An eight channels brain system electronics (Image Guided Therapy, Pessac, France) was used to operate a magnetic resonance (MR) compatible phased array annular transducer operated at a central frequency of 1.5 MHz (diameter 30 mm, geometrical focal depth 20 mm; Imasonic, Voray sur l’Ognon, France). The annular transducer allowed steering of the ultrasound beam in the antero-posterior direction. Head/foot and left/right translations were motorized HR4 and HR2 motors respectively (Nanomotion, Yoqneam, Israel). The rod holding the transducer could be manually lift up and down and the transducer horizontality was manually corrected using a spirit level. Coupling was insured by a water balloon between the transducer and the animal head. A degassing system allowed circulation of degassed, deionized water in the balloon prior to the experiments. The volume of water in the balloon could be adjusted manually after positioning the transducer above the head to ensure a proper coupling.

*MRI acquisitions.* All MR procedure was performed on a 11.7T Biospec 117/16 (Bruker Biospin, Germany) running Paravision 6.0.1 with a maximum gradient amplitude of 750 mT/m. MR sequences were taken before and after the BBB disruption procedure using Gadolinium contrast agent to ensure that BBB was well opened on the predefined zone. An IGT (Image Guided Therapy, France) transceiver was used for both signal emission and reception. This consisted in a U-shape MR coil resonating around 500 MHz. To define the targeted zone of BBB disruption, three sets of twenty contiguous 1 mm slices were successively acquired with a FLASH sequence on coronal, sagittal and axial planes. Parameters were: TR/TE = 300/4 ms, Flip angle = 60°, Bandwidth = 50 kHz, 1 average, Matrix size = 256x256 for a 0.20 mm in plane resolution. Total scan time was 4 minutes for the whole targeting procedure. After this initial step followed by a Gadolinium injection into the tail vein, a first 2D Fat Saturated Spin Echo sequence was acquired to check for BBB integrity. Parameters were: TR/TE = 200/5.2 ms, Bandwidth = 50 kHz, 6 averages, Matrix size = 128x128 for a 0.15 mm in plane resolution. Total scan time was 2.5 min. This MR sequence was repeated after the BBB disruption procedure.

*BBB disruption.* Adult C57Bl/6 mice (n=3) were anaesthetized with isoflurane (1-2% at beginning, then 0.8% during MRI acquisition). The catheter was installed in the tail vein and the head was shaved with a depilatory cream. Mice were then installed on the MRgFUS system (Image Guided Therapy) and the transducer was placed on their head. MR contrast agent (MRCA) (Dotarem, Guerbet, France; 0.1 mL) was injected intravenously outside the MRI tunnel, before the baseline MRI acquisition. Mice were then removed from the MRI tunnel in order to inject the ultrasound contrast agent (UCA) (Sonovue, Bracco, Italy; 0.15 mL). Focused Ultrasound (FUS) was applied continuously on a trajectory on the brain right hemisphere during 150 s (200 trajectories with 50 ms pause between them) at 4% amplitude, corresponding to an estimated derated pressure of 0.35 MPa in the mice brain. FUS onset was synchronized with UCA injection, the latter lasting about 10 s. MRCA was then injected (0.12 mL) before MRI acquisition. The FUS sonication took place 1 h 30 mins after anaesthesia. The entire procedure, from anaesthesia to OCT injection, lasted about 2 h.

***Cerebral ischaemia****.* Isoflurane-anaesthetized P14 rats were exposed to proximal left middle cerebral artery electrocoagulation (MCAo) combined with a transient (60 min) double occlusion of common carotid arteries (CCAo). Carotid blood-flow restoration was assessed by release of the carotid clips.

***Traumatic brain injury***. In isoflurane-anaesthetized P7 mice, the skull surface was exposed and the foot-plate (2 mm diameter) of the impact device was positioned 2 mm anterior and 1 mm lateral to lambda on the left cranial side. First the foot-plate was allowed to touch the skull and then was depressed by 0.5 mm. The contusion impact was delivered by a 10 g weight falling onto the foot-plate from a height of 10 cm.

**OCT transport assays through hPBMECs monolayers.** The hPBMECs were isolated from surgical resections of a brain tumour patient (55-year-old male suffering from a grade III oligodendroglioma). The experimentation was conducted in compliance with the French legislation, and the protocol was permitted by the French Ministry of Higher Education and Research (CODECOH DC-2014-2229). Brain capillaries were isolated using soft digestion of patient brain tissues and then seeded. Brain primary microvascular endothelial cells were shortly amplified and seeded on 12-well-type Transwell® (Corning, membrane area: 1.12 cm^2^) with microporous membranes (pore size: 0.4 µm). Cells were cultured in EBM-2 medium (Lonza, Basel, Switzerland) supplemented with 20% serum and growth factors (Sigma-Aldrich, St. Louis, MO, USA). Transendothelial electrical resistance (TEER) was measured as an integrity marker and to assess cellular barrier tightness.

***Immunocytochemistry****.*

*Immunofluorescence.* Sections were preincubated in PBS with 5% normal donkey serum (NDS; Sigma-Aldrich) and 0.3% Triton X-100 for 30 mins at RT, incubated in primary antibodies diluted in PBS with 1% NDS and 0.3% Triton X-100 overnight at RT, rinsed in PBS, and incubated in fluorescent secondary antibodies in PBS with 3% NDS and 0.3% Triton X-100 for 1 h at RT. Finally, sections were rinsed in PBS, stained with DAPI 1:1000 in PBS for 2 mins at RT, rinsed in PBS, mounted on glass slides and coverslipped with Fluoromount (SouthernBiotech, Birmingham, AL, USA) for confocal microscopic analysis. The same immunofluorescent procedure was used on the DAOY cells of the *in vitro* model. Sequential immunostaining was applied for SST2 and GLUT-1 double-labelling to minimize cross-reactivity of secondary antibodies.

*Chromogenic labelling.* For volume estimation of SST2 agonist extravasation in the *in vivo* model 2, free floating mouse brain sections were processed for SST2 immunohistochemistry using the immunoperoxidase method. Endogenous peroxidase activity was quenched by incubating the sections in 0.3% H_2_O_2_ in PBS for 30 min. After rinsing in PBS, sections were preincubated in PBS with 5% normal goat serum (NGS; Sigma-Aldrich) and 0.3% Triton X-100 for 30 mins at RT, incubated in anti-SST2 antibody (Abcam) diluted in PBS with 1% NGS and 0.3% Triton X-100 overnight at RT, rinsed in PBS, incubated in biotinylated goat anti-rabbit antibody (Vector Laboratories) diluted in PBS with 3% NDS and 0.3% Triton X-100 for 1 h at RT, rinsed in PBS, and incubated in avidin-biotinylated horseradish peroxidase complex (ABC; Vector Laboratories) diluted 1:200 in PBS for 90 mins at RT. After repeated washing in PBS and then in 0.05 M Tris buffer, pH 7.4 (TB), peroxidase activity was revealed with 0.05% of 3,3'-diaminobenzidine (DAB; Sigma-Aldrich) in TB, in the presence of hydrogen peroxide (0.0048%). The reaction was stopped by several washes in TB. Sections were mounted on gelatin-coated slides, dehydrated in graded ethanols, delipidated in xylene and coverslipped with Permount (Fisher Scientific, Pittsburgh, PA, USA) for light microscopic observation.

***Confocal microscopy.*** Eight-bit digital images were collected from a single optical plane using a 20x HC PL APO CS2 oil-immersion Leica objective (numerical aperture 0.75) or a 63x HC PL APO CS2 oil-immersion Leica objective (numerical aperture 1.40). For each optical section, double- or triple-fluorescence images were acquired in sequential mode to avoid potential contamination by linkage specific fluorescence emission cross-talk. Settings for laser intensity, beam expander, pinhole (1 Airy unit), range property of emission window, electronic zoom, gain and offset of photomultiplicator, field format, scanning speed were optimized initially and held constant throughout the study so that all sections were digitized under the same conditions. Images were equally adjusted for brightness and contrast, and composite illustrations were built in Adobe Photoshop CS3 (Adobe Systems, San Jose, CA, USA).

For three-dimensional (3D) imaging, Z-stacks with a step of 346 nm between consecutive optical sections using a 40x HC PL APO CS2 oil-immersion Leica objective (numerical aperture 1.30) and with a step of 300 nm between consecutive optical sections using a 63x HC PL APO CS2 oil-immersion Leica objective (numerical aperture 1.40) were collected. 3D reconstructions and videos were built with the Leica Laser Application Suite X software (Leica Microsystems).

***Electron microscopy.*** For pre-embedding immunogold immunocytochemical detection of SST2 at the electron microscopic level, mice were deeply anaesthetized with sodium pentobarbital (150 mg/kg i.p.) and perfused through the ascending aorta with 100 mL of ice-cold 4% PFA and 0.05% glutaraldehyde (GA) in PB. Brains were post-fixed overnight in 4% paraformaldehyde at 4°C. Coronal sections were cut on a vibratome at 70 µm and collected in PBS. Sections were equilibrated in 25% sucrose and 10% glycerol in 0.05 M PB, frozen rapidly in isopentane cooled in liquid nitrogen, and thawed in PBS at RT. Sections were preincubated for 30 mins in 5% NGS in PBS, and then incubated overnight at RT in rabbit anti-SST2 antibody (1:1000) diluted in PBS containing 1% NGS. After washing in PBS, sections were incubated for 120 mins in Nanogold®-conjugated goat anti rabbit IgG (1:100; Nanoprobes, Yaphank, NY, USA) diluted in PBS containing 2% of bovine serum albumin-c and 0.2% of cold water fish gelatin. Sections were washed in PBS and post-fixed in 1% GA in PBS for 10 min. After washings in PBS and 0.1 M sodium acetate buffer, pH 7.0, SST2 immunogold labelling was intensified using a silver enhancement kit (HQ Silver; Nanoprobes) for 5-10 mins in the dark at RT. After washings in acetate buffer and in PB, sections were post-fixed in 1% osmium tetroxide in PB for 10 mins at RT. After washings in PB, they were dehydrated in an ascending series of ethanol, which included 1% uranyl acetate in 70% ethanol. They were then treated with propylene oxide twice for 10 min, equilibrated overnight in Durcupan ACM (Fluka, Buchs, Switzerland), mounted on glass slides and cured at 60°C for 48 h. Areas of interest were cut out from the slide and glued to blank cylinders of resin. Blocks were cut in semithin sections (1 µm) and then in ultrathin sections on a Reichert Ultracut S microtome. Ultrathin sections were collected on pioloform-coated single-slot grids. Sections were stained with lead citrate and examined with a Philips CM120 electron microscope equipped with Morada Soft Imaging System (Olympus Soft Imaging Solutions, Munster, Germany).

**
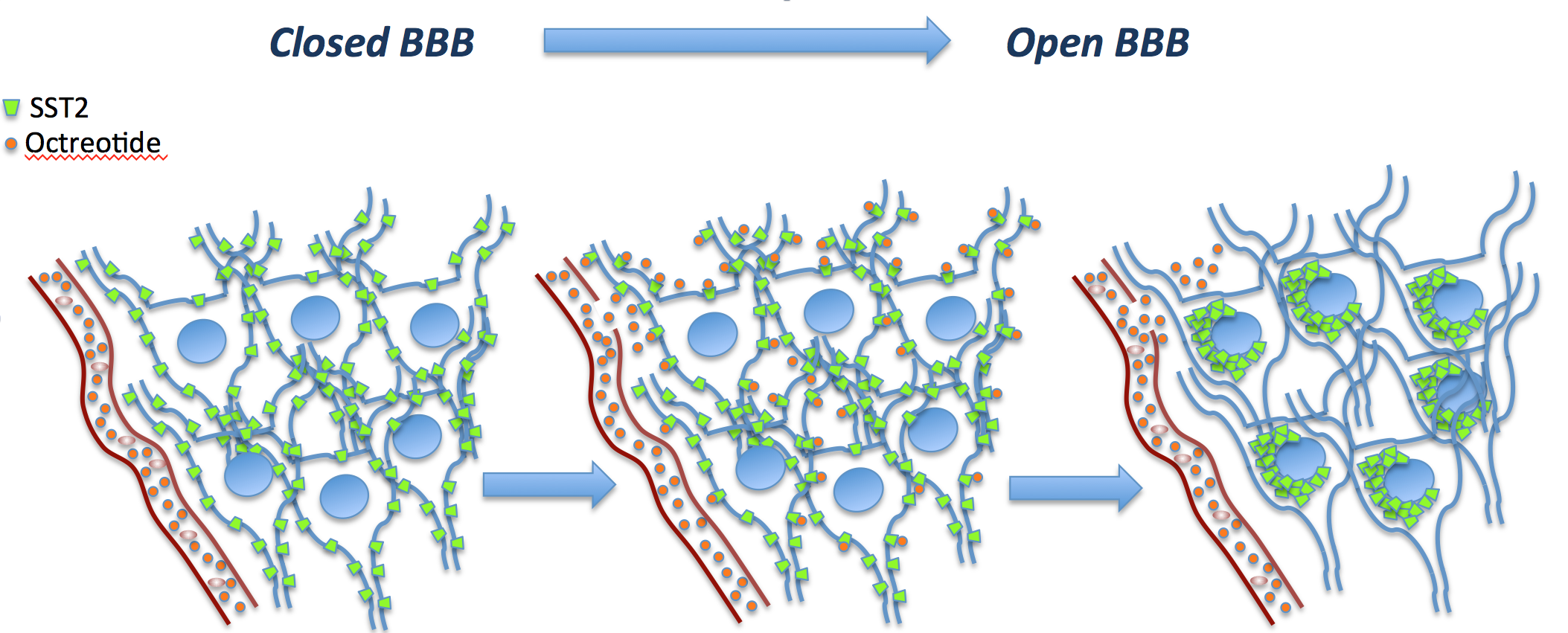
**

**Figure S1. Schematic representation of detection of increased blood-brain barrier (BBB) permeability using ligand-induced somatostatin receptor 2 (SST2) internalization.** Following leakage of the BBB, octreotide injected intraperitoneally diffuses from blood vessels into the brain parenchyma and subsequently binds to surface SST2 of adjacent neurons. Agonist binding leads to receptor internalization and clustering in the *trans-*Golgi network. This change in receptor distribution from cell surface to intracellular leads to an unambiguous characterization of vascular leakage in the brain. Blue lines, neuronal plasma membrane; blue circles, neuronal nuclei.


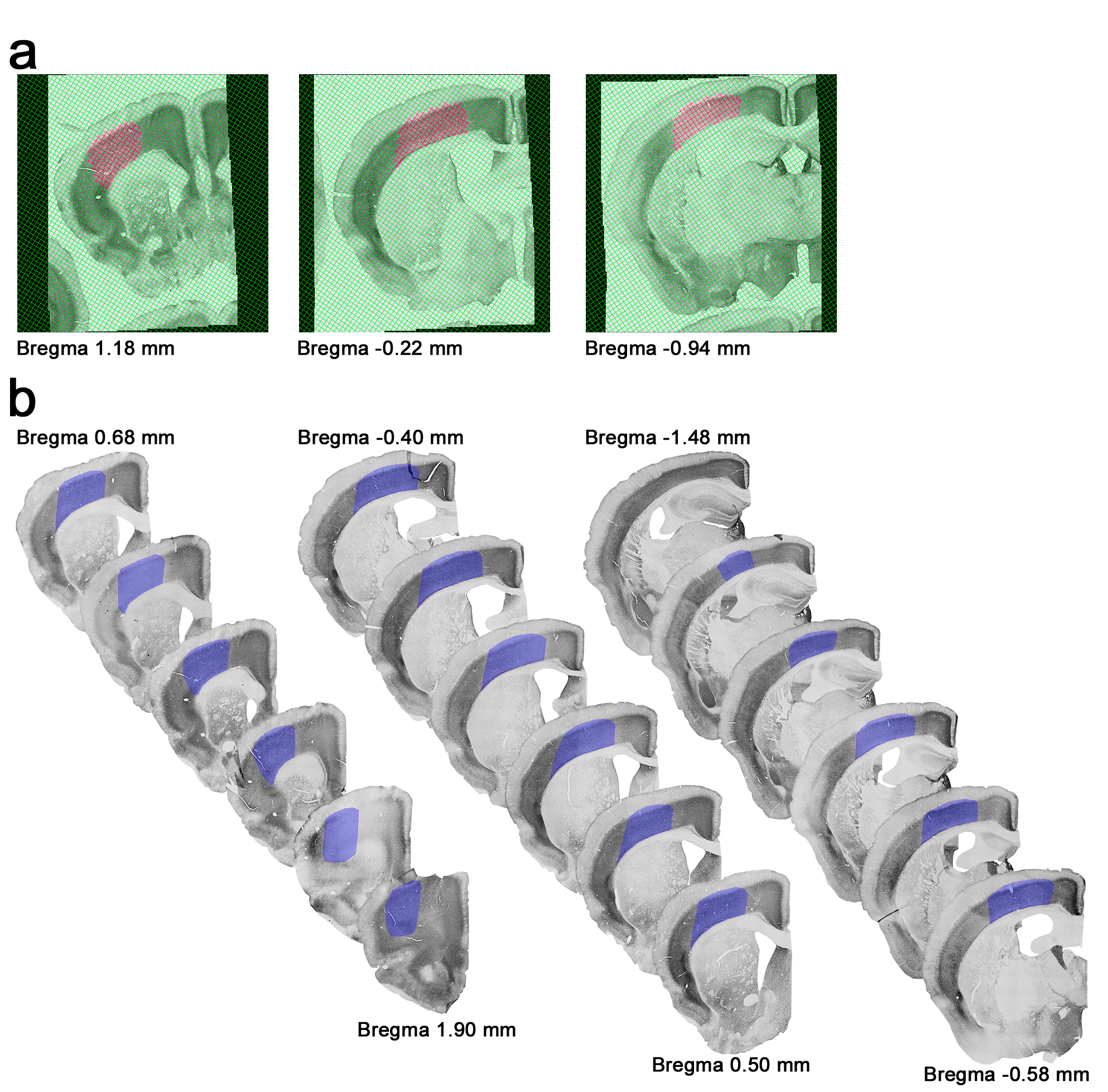


**Figure S2. Quantification of somatostatin receptor 2 (SST2) agonist extravasation after focused ultrasound-mediated (MRgFUS) disruption of the blood-brain barrier (BBB) in the cerebral cortex.** In the FUS-targeted right cerebral cortex, area of agonist-induced SST2 internalization is manually traced and the volume estimate is calculated using the Volumest ImageJ plug-in (a; 3 representative rostro-caudal levels). Serial sections illustrate the extent of BBB disruption detected by agonist-induced SST2 internalization (shaded areas on b). The volume of the BBB disruption was estimated as 3.59x10^9^ μm^3^.

**
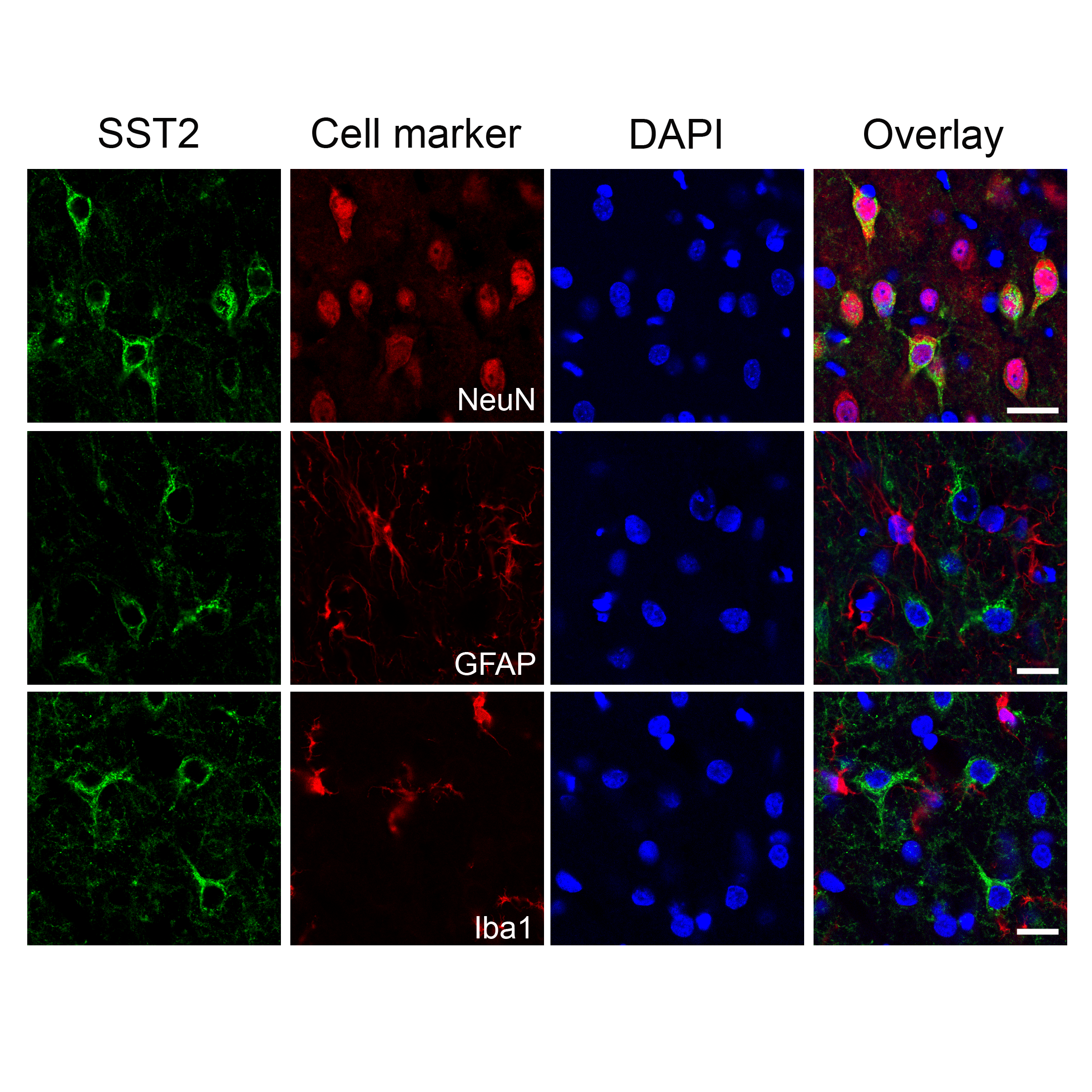
Figure S3. Characterization of cells with internalized somatostatin receptor 2 (SST2) following SST2 agonist extravasation in the rodent brain.** Somatodendritic SST2-immunoreactive profiles characteristic of agonist-induced internalization colocalize with neuronal marker NeuN, as illustrated in the juvenile cerebral ischaemia rat model. Note, that all SST2-positive cells are also NeuN-immunoreactive, conversely the majority of NeuN-positive cells are SST2-immunoreactive. No colocalization of SST2-labelled cells with astrocyte marker GFAP or microglia marker Iba1 is detected. Scale bars: 20 μm.

**
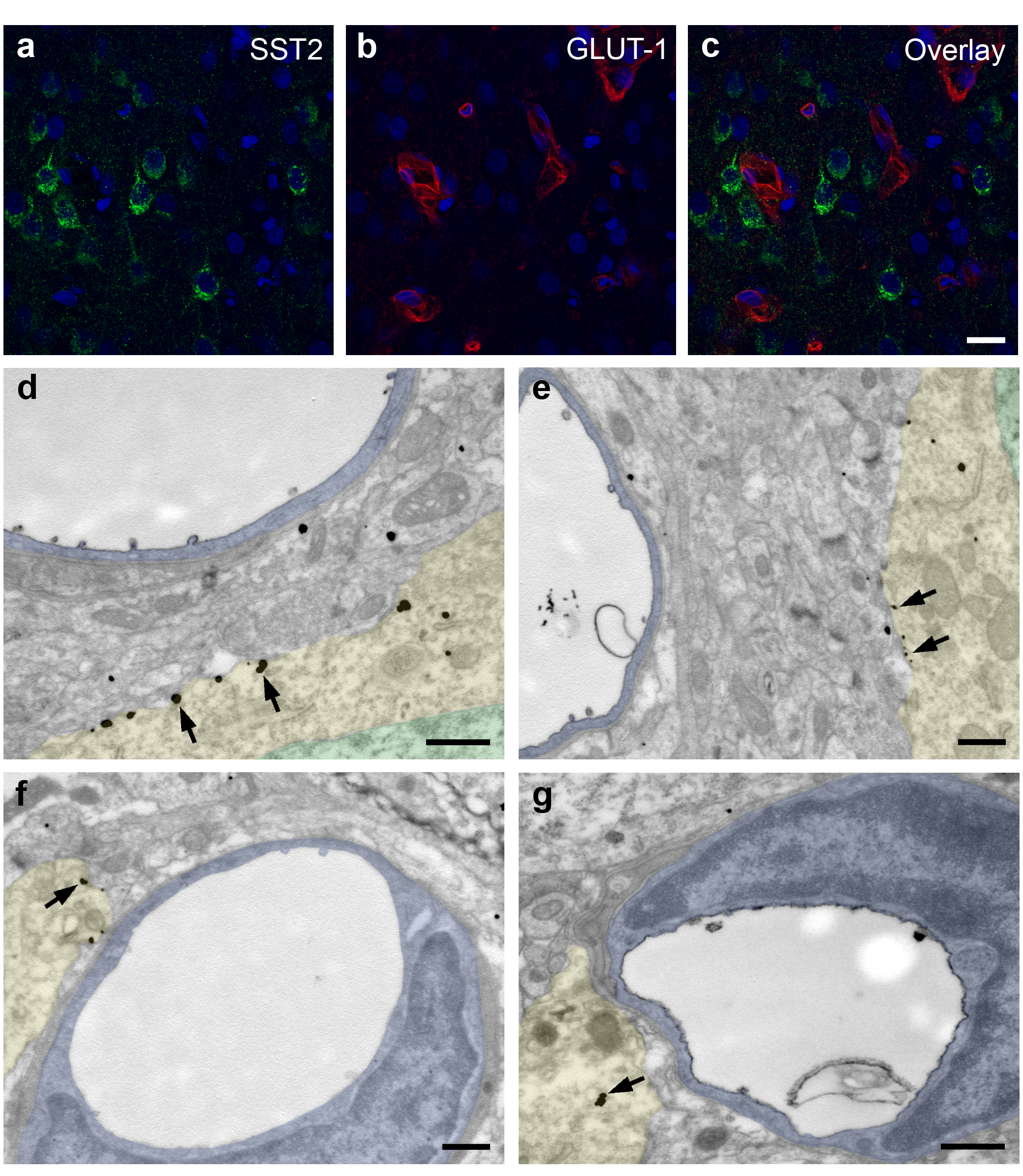
**

**Figure S4. Characterization of somatostatin receptor 2 (SST2) localization in relation to blood vessels in the rodent brain.** At the light microscopic level, no colocalization is detected between the somatodendritic SST2-immunoreactive profiles following agonist-induced internalization and the GLUT-1-immunoreactive blood vessels (a-c), as illustrated in the juvenile cerebral ischaemia rat model. Nuclei are labelled with DAPI. At the electron microscopic level, SST2 immunoparticles are localized in neuronal perikarya and dendrites (yellow shaded area on d-g), as illustrated in the mouse cerebral cortex. Immunoparticles are associated with the plasma membrane (d-f) (arrows) as well as with endosomes (g) (arrow). No SST2 immunoparticles is detected at the plasma membrane or in the cytoplasm of endothelial cells (blue shaded area on d-g). Green shaded area, nucleus. Scale bars: a-c, 20 μm, d-g, 0.5 μm.


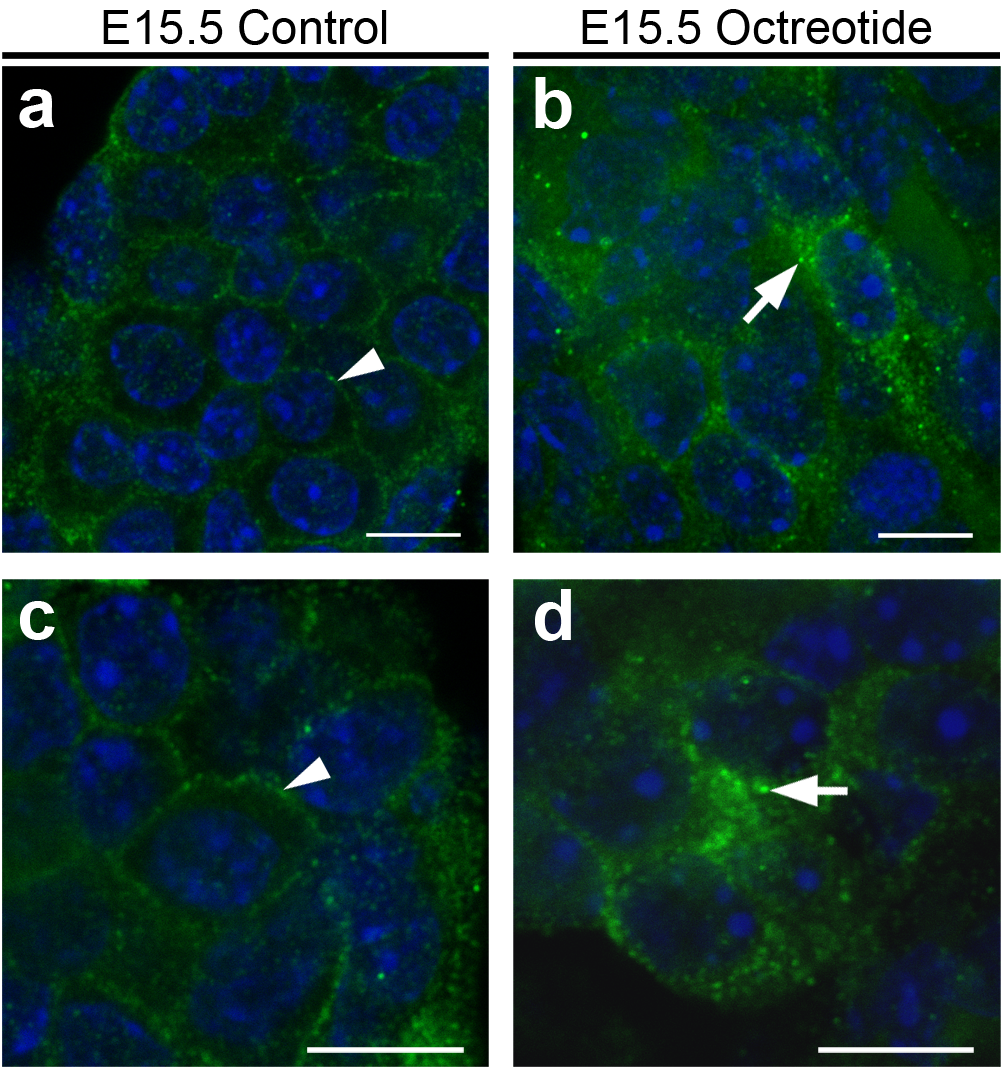


**Figure S5.** **Confocal microscopic analysis of somatostatin receptor 2 (SST2) immunoreactivity in the periphery of E15.5 embryos following SST2 agonist injection of the dams.** In a control E15.5 embryo, SST2 immunoreactivity is predominantly localized at the surface of endocrine pancreatic cells (a, c) (arrowheads). Following i.p. injection of octreotide in the pregnant dams, SST2 immunoreactivity is concentrated in bright intracellular fluorescent granules in the pancreas of a E15.5 embryo (b, d) (arrows). Nuclei are labelled with DAPI. Scale bars: a, b, 7 μm; c, d, 8 μm.

**Video S1.** **Low magnification 3D reconstruction** **of somatostatin receptor 2 (SST2) immunoreactivity in the mouse cerebral cortex following focused ultrasound-mediated (MRgFUS) opening of the blood-brain barrier (BBB).** In the FUS-targeted right cerebral cortex, somatodendritic SST2-immunoreactive profiles characteristic of agonist-induced internalization is detected following i.p. injection of SST2 agonist octreotide (right panel) as compared to the homogenous SST2 labelling in the surrounding cortex (left panel).

**Video S2. High magnification 3D reconstruction of somatostatin receptor 2 (SST2) immunoreactivity in the mouse cerebral cortex following focused ultrasound-mediated (MRgFUS) opening of the blood-brain barrier (BBB).** Within the FUS-targeted area, bright intracytoplasmic SST2 immunofluorescent granules are evident around blue DAPI-stained nuclei following i.p. injection of SST2 agonist octreotide (right panel). In the surrounding control cortex, a dense meshwork of SST2 immunofluorescence is located in the neuropil (left panel).
